# Convection-enhanced delivery of nanoencapsulated gene locoregionally yielding ErbB2/Her2-specific CAR-macrophages for brainstem glioma immunotherapy

**DOI:** 10.1186/s12951-023-01810-9

**Published:** 2023-02-20

**Authors:** Lin Gao, Chongdeng Shi, Zhenmei Yang, Weiqiang Jing, Maosen Han, Jing Zhang, Cai Zhang, Chunwei Tang, Yuanmin Dong, Ying Liu, Chen Chen, Xinyi Jiang

**Affiliations:** 1 NMPA Key Laboratory for Technology Research and Evaluation of Drug Products and Key Laboratory of Chemical Biology (Ministry of Education), School of Pharmaceutical Sciences, Cheeloo College of Medicine, Shandong University, 44 Cultural West Road, Jinan, 250012 China; 2 Department of Urology, Qilu Hospital, Cheeloo College of Medicine, Shandong University, 107 Cultural West Road, Jinan, 250012 China

**Keywords:** Brainstem gliomas, Macrophages, Immunotherapy, Phenotypic transformation, CAR

## Abstract

Locoregional delivery of chimeric antigen receptor (CAR)-modified T (CAR-T) cells has emerged as a promising strategy for brain tumors. However, the complicated ex vivo cell manufacturing procedures and the rapid progression of the disease have limited its broader applications. Macrophages (MΦs) exhibit unique effector functions and a high degree of infiltration within the solid tumor microenvironment (TME), especially in the brain, where MΦs function as structural support, and the main immune effector cells of the CNS represent 5–12% of brain cells. Here, we report a synthetic universal DNA nanocarrier for in situ genetic editing of intratumoral MΦs with an ErbB2-specific CAR to direct their phagocytic activity towards tumors and subsequently initiate a locoregional antitumor immune response. Specifically, we demonstrated that when delivered locoregionally, the RP-182 peptide, located in the shell of a nanoparticle, targeted MΦs and reprogrammed M2-like tumor-associated macrophages (TAMs) to an antitumor M1-like phenotype. Subsequently, the CAR gene-laden DNA nanocomplex can be used to introduce ErbB2-targeted CAR, and the generated CAR-MΦs then act as “living” cures, thereby serially clearing the invasive tumor cells. Our work demonstrates a practical antitumor immunotherapy for brainstem gliomas (BSGs) that may be broadly applicable for patients suffering from other ErbB2-positive solid malignancies.

**Supplementary Information:**

The online version contains supplementary material available at 10.1186/s12951-023-01810-9.

## Introduction

Brainstem gliomas (BSGs), which predominantly affect the central nervous system (CNS) of children, represent a formidable challenge in the clinic with a grim prognosis [1–3]. Despite modern developments in aggressive multimodal therapies, the outcome for children with BSGs remains quite poor [4–6]. The major challenges in the treatment of BSGs are its extremely invasive nature and delicate anatomical location in the brain stem [7], which precludes surgical removal, and BSG cells demonstrate drug resistance to chemotherapeutics [8–10]. Tracking and eradicating invasive tumor cells is of the utmost importance for preventing these devastating diseases, yet effective strategies remain elusive.

Despite chimeric antigen receptor (CAR)-modified T (CAR-T) cells have established clinical efficacy against B-cell malignancies [11, 12], BSG patients do not respond to the current state-of-the art therapies [13, 14]. One of the main factors contributing to poor response to CAR-T treatment is the incompetence of CAR-T cells to reach and survive in the tumor immune microenvironment (TME) [15–17]. In contrast to T lymphocytes, macrophages (MΦs) are one of the most abundant nonneoplastic cell types in the brain TME, accounting for approximately 30% of the tumor composition [16, 18–20]. Insights regarding the importance of MΦs in the TME has initiated interest in therapeutic approaches to reverse the functions of immunosuppressive tumor-associated macrophages or to heighten MΦ phagocytosis [21–24]. The importance of macrophages in the TME has aroused interest in therapeutic efforts to remodel the immunosuppressive phenotype or disinhibit the phagocytic activity of tumor-supportive macrophages. One recent study indicate that intratumoral macrophages could be engineered with tumor-specific CARs, and the engineered CAR macrophages (CAR-Ms) were found to display enhanced phagocytic activity toward cancerous cells and thus reduced the tumor burden and prolonged overall survival in mouse models [25]. Considering their unique effector functions and the abundance of MΦs in the brain [19, 26], we hypothesized that genetic programming of the intratumour MΦs with BSG-specific CAR could redefine their phagocytic activity and antigen presenting functions to stimulate an adaptive immune response against BSG, thus promoting tumor remission.

ErbB2, also known as human epidermal growth factor 2 (HER2), is an appealing target for the treatment of BSG [27, 28]. As shown in Additional file 1: Figure S1A, B, the expression level of the ErbB2 protein in tumor tissues was found to be obviously higher than that in normal tissues in BSG patients. Multiple ErbB2-targeted therapeutics, such as trastuzumab (Herceptin), present clinical success in patients with multiple ErbB2 + tumor types [29, 30]. As such, ErbB2 may be a critical target for BSG treatment [31]. Therefore, we hypothesized that tumor-supportive macrophages genetically engineered in situ with an ErbB2-specific CAR could exhibit enhanced phagocytic activity against cancerous BSG cells and subsequently initiate locoregional antitumor immunity.

**Fig. 1:**
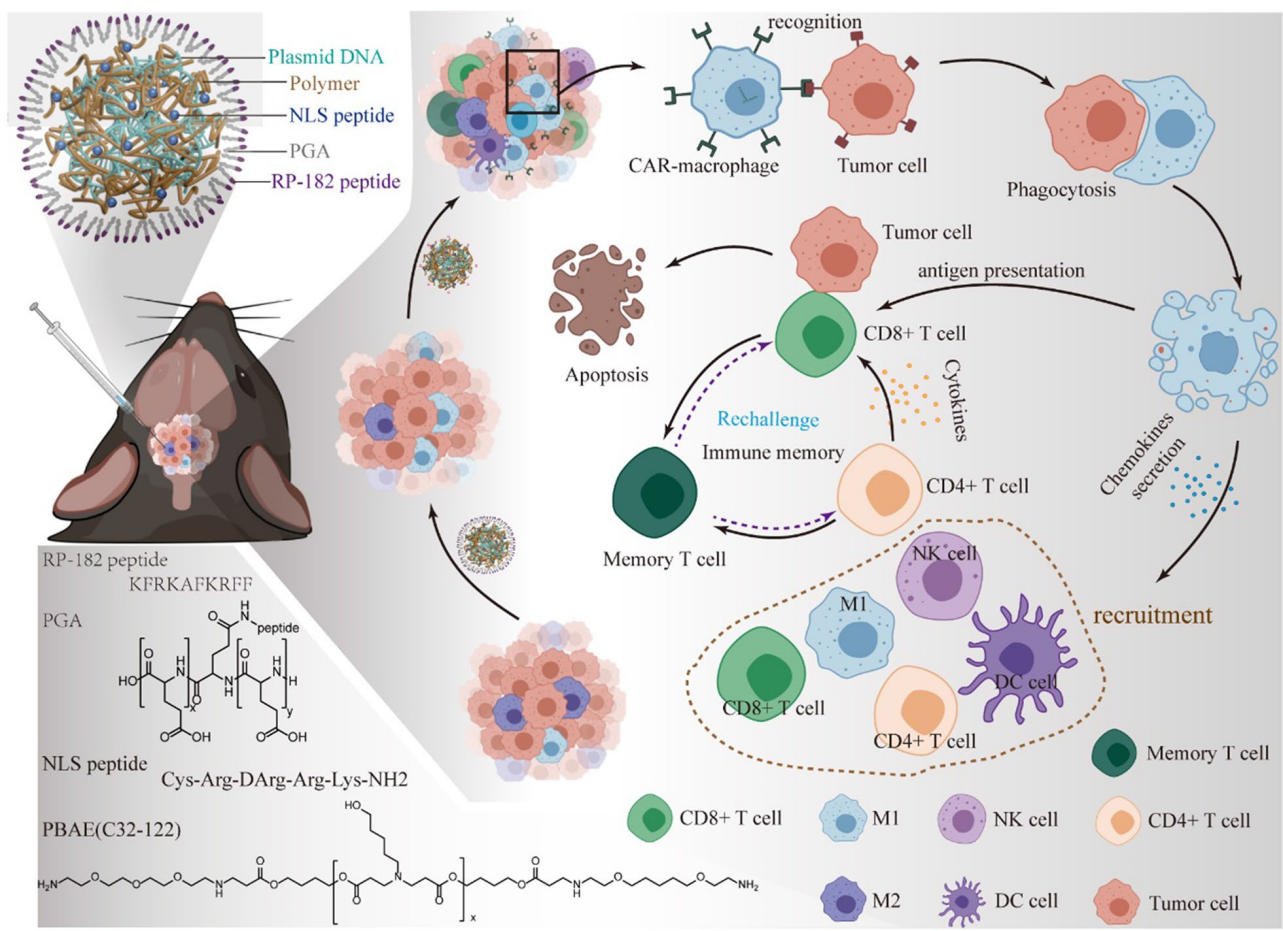
Design and therapeutic mechanisms of macrophage-programming nanoparticles. Additionally, the chemical structures of the PBAE C32-122 polymer and polyglutamic acid are shown, as well as the amino acid sequence of the nuclear localization (NLS) peptide

Currently, manufacturing CAR editing cells, including CAR-T cells and CAR- natural killer (NK) cells ex vivo, requires an assortment of elaborate protocols to isolate, genetically modify, and selectively expand the redirected cells before they are infused back into the patient [32–35]. The requirements for dedicated equipment and considerable technical expertise and complicated and costly procedures pose challenges for broader clinical application [36–38]. Herein, we proposed the establishment of a synthetic DNA nanocarrier that can quickly and specifically program tumor-recognizing capabilities into macrophages in the TME. Moreover, RP-182 peptides, the synthetic 10-mer amphipathic analog of host defense peptides, were used as a nanoshell to assist reprogramming M2-like tumor-associated macrophages (TAMs) to an antitumor M1-like phenotype [39]. RP-182 peptide activates phagocytosis and autophagy in M2-like macrophages via the mannose receptor CD206, reverting these cells into an antitumor M1-like phenotype with increased M1 cytokine production, as well as the ability to phagocytose cancer cells and to aid tumor antigen recognition of intratumoral CD8 + T cells. Both activation of autophagy and NF-κB signaling, together with metabolic reprogramming of M2-like macrophages, have previously been shown to transform M2 macrophages toward an M1 phenotype [40, 41]. Meanwhile, RP-182 induces apoptosis via cleaved caspase 8 and an autocrine-positive feedforward loop involving TNF-α signaling, promoting the depletion of this population and further shifting the balance toward the proinflammatory, antitumor M1 phenotype [39]. In addition, RP-182 peptide and the CAR plasmid both help macrophage phagocytose cancer cells and to aid tumor antigen recognition of intratumoral CD8 + T cells [42]. This approach is convenient and cost-effective; additionally, with this strategy, DNA nanocarriers act as a “living” cure to repolarize TAMs toward an M1 phenotype, and serially, the macrophages are programmed into CAR-MΦs. Then, CAR-MΦs engulf tumor cells and draw in T and NK cells, which recruits antigen presenting cells (APCs), notably dendritic cells (DCs), and leads to a broad adaptive immune response that has the potential to generate long-term immunity (Fig. 1).

## Results and discussion

### Design and manufacture of macrophage-programming nanoparticles

**Fig. 2:**
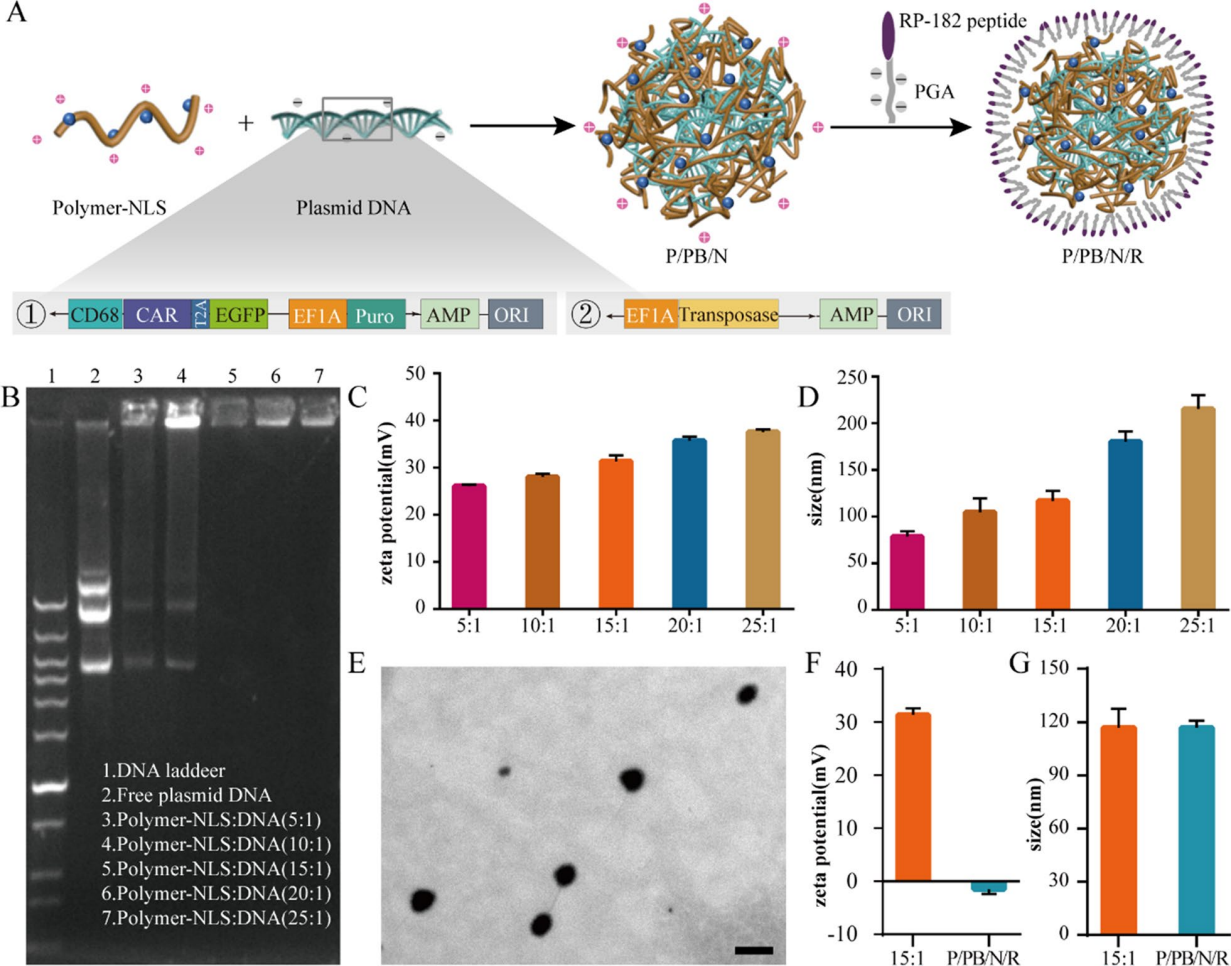
Design and characterization of the nanocarriers. **A** Schematic illustration of the preparation of nanocarriers (top) and schematic of the ErbB2-CAR plasmid (bottom). **B** Agarose gel retardation assay of the CAR plasmid at various weight ratios. **C** Zeta potentials and **D** diameters of nanoparticles containing plasmid DNA with various weight ratios. **E** Transmission electron microscopy (TEM) image of representative P/PB/N/R nanoparticles. Scale bar, 200 nm. **F** Zeta potentials and **G** diameters of nanoparticles containing plasmid DNA with 15:1 weight ratios and P/PB/N/R nanoparticles

Effective nucleic acid expression by macrophages depends on the cellular uptake of nanoparticles by macrophages and importation of DNA cargo into the cell nucleus [23, 42]. The first step we performed was to couple macrophage-cell-targeting and phenotype-switching RP-182 peptide onto the surfaces of biodegradable poly (β-amino ester)-based nanoparticles. Then, we functionalized the polymer with peptides containing nuclear localization sequence to facilitate fast-track nuclear import of their genetic cargo (Fig. 2A). Containing the high positive charge at physiological pH and the fact that the NLS sequence was arginine- and lysine-rich, the nuclear localization peptide might induce that DNA aggregating behaviour could be hypothesized [43]. Electrostatic forces indeed play an important role in the interactions of small molecules and proteins with nucleic acids, due to the polyanionic nature of the latter. Arginine and lysine groups in peptides and proteins are heavily involved in H-bonds in DNA–protein complexes. Most of the small molecule compounds targeting nucleic acids carry multiple positive charges as well [44]. Thus, modification of the nuclear localization peptide can improve the transfection efficiency of nanoparticles into macrophages.

We furnished these targeted nanoparticles with anticancer programming capabilities by loading them with plasmid DNA encoding the ErbB2-specific CAR with the CMV promoter or macrophage specific CD68 promoter (Fig. 2A and Additional file 1: Figure S2–4), which was reported to be capable of driving specific gene expression in macrophages [45].

Hence, we developed nanoparticles through the electrostatic interactions between the CAR plasmid and polymer to locoregionally yield ErbB2-specific CAR-MΦs in the tumor site. As shown via agarose gel electrophoresis, the DNA plasmid was completely condensed within the polymer nanoparticles when the weight ratio of polymer and plasmid in nanoparticles was greater than 15:1 (Fig. 2B). In addition, the surface charge (Fig. 2C) and size (Fig. 2D) of the nanocomplex both increased as the ratio changed from 5:1 to 25:1. Considering the plasmid compression factor, we developed P/PB/N/R nanoparticles with a 15:1 mass ratio. RP-182 peptide conjugated with polyglutamic acid (PGA) was coated onto the outer layer to achieve macrophage cell targeting. The P/PB/N/R nanocarrier exhibited a well-defined spherical structure under transmission electron microscopy (TEM) (Fig. 2E) with an average diameter of 110.3 nm (Fig. 2G). In addition, the zeta potential of the P/PB/N/R nanocarrier was − 1.48 ± 0.9 mV (Fig. 2F). And to evaluate stability of the nanocomplexes in serum, P/PB/N/R nanoparticles were incubated in 50% serum and its size was measured 7 days, which exhibited excellent stability (Additional file 1: Figure S5).

### *CAR-programming of macrophages *via* DNA nanocarriers*

**Fig. 3:**
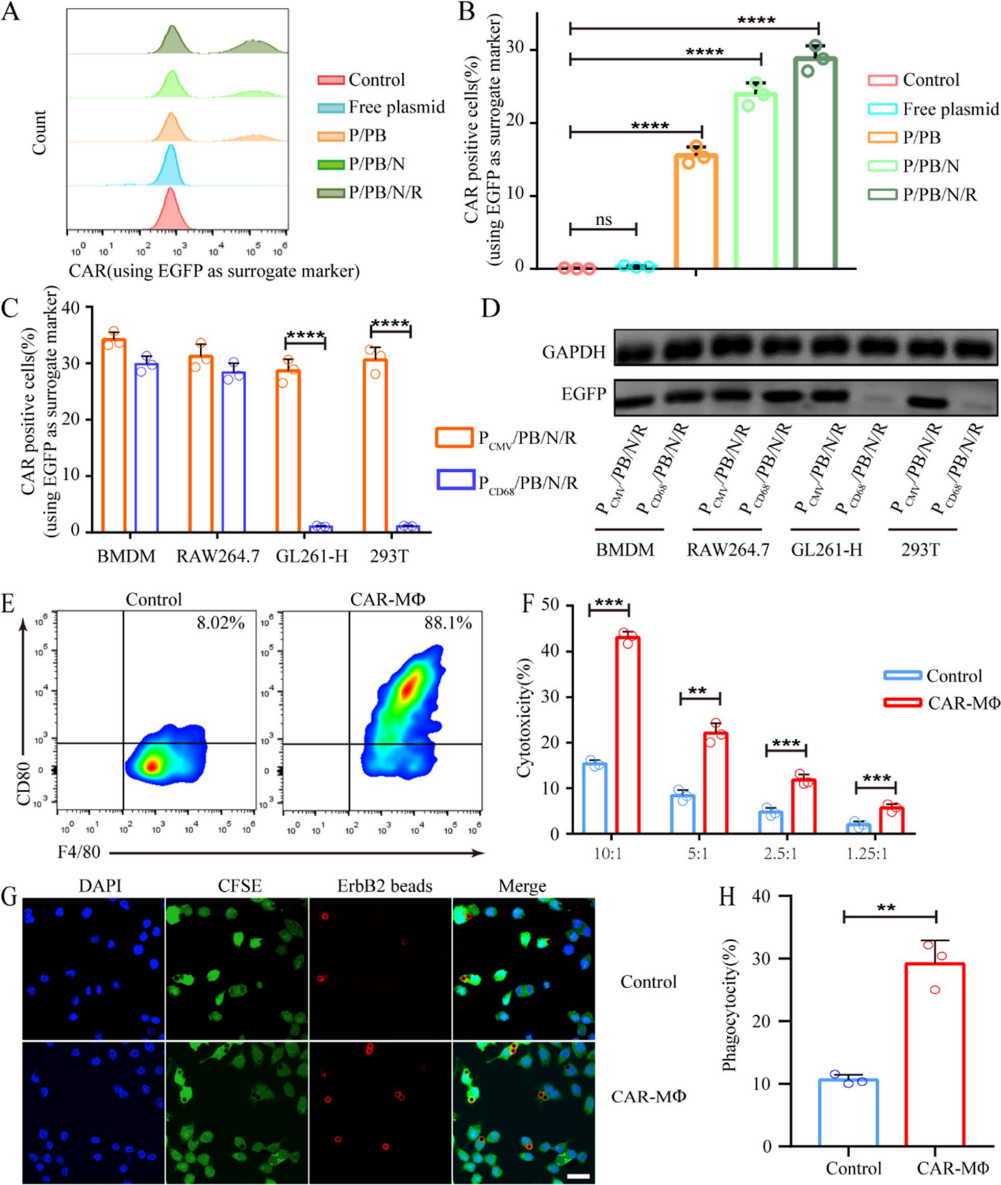
CAR expression and effector activity of CAR-MΦs in vitro. **A** Percentages of CAR-positive RAW264.7 cells after treatment with various nanoformulations (phosphate-buffered saline (PBS), free plasmid, P/PB, P/PB/N, P/PB/N/R; P/PB contains plasmid DNA and PBAE C32-122, P/PB/N contains plasmid DNA and PBAE C32-122-NLS, and P/PB/N/R contains plasmid DNA, PBAE C32-122-NLS and PGA–RP-182 peptide. PBS and free plasmid were used as control groups). **B** Data are presented as a histogram and percentages. Data are shown as the mean ± SEM (n = 3). **C** CAR expression in BMDMs, RAW264.7 cells, GL261-H cells and 293 T cells after treatment with P_CD68_/PB/N/R nanoparticles or P_CMV_/PB/N/R nanoparticles determined by flow cytometry and **D** Western blotting. **E** Flow cytometry analysis of the M1-related marker CD80 in RAW264.7 (left) and CAR-MФ (right) cells. **F** LDH cytotoxicity assay of CAR-MФs. Cytotoxicity was evaluated at different effector: target cell ratios in GL261-H cells. Data are representative of three independent experiments. **G** Confocal images and **H** statistical graph of phagocytotic ability of RAW264.7 and CAR-MФ for the uptake of ErbB2 beads. Images from left to right show DAPI-stained cell nuclei (blue), CFSE-stained cells (green), ErbB2 beads (red), and merged images. Data are shown as the mean ± SEM (n = 3). Statistical significance was calculated via a two-tailed Student’s t test. **P < 0. 01; ***P < 0.001; ****P < 0.0001

Then, we assessed the capacity of the P/PB/N/R nanoparticles to program macrophages by incubating them with RAW264.7 cells. As shown in Fig. 3A, B, the expression of CAR mediated by P/PB/N/R nanoparticles exhibited the highest level of all the tested nanoformulations, while the cellular uptake of P/PB, P/PB/N and P/PB/N/R nanoparticles was comparable (Additional file 1: Figure S6). These results confirmed the superior transfection efficiency of P/PB/N/R nanoparticles mediated by NLS and RP-182 peptide. To validate the specific CAR expression, which used the CD68 promoter, in macrophages, we manufactured P_CD68_/PB/N/R and P_CMV_/PB/N/R nanoparticles, which contained plasmids with CD68 and CMV promoters, respectively. And the P_CD68_/PB/N/R and P_CMV_/PB/N/R nanoparticles were utilized to edit different cells, including bone-marrow-derived macrophage (BMDMs) and RAW264.7, 293 T and GL261-H cells. After the cells were modified by the P/PB/N/R with different plasmid promoters, the expression levels of the CAR were measured. As shown in Fig. 3C, all cells exhibited comparable CAR expression when transfected with P_CMV_/PB/N/R. The same result was observed for murine-derived BMDMs and RAW264.7 cells, while nearly no CAR expression was observed in 293 T and GL261-H cells when transfected with P_CD68_/PB/N/R. The result was further confirmed by the western blot assay results provided in Fig. 3D and Additional file 1: Figure S7. These data demonstrate the superiority of the CD68 promoter for macrophage gene editing. Furthermore, high levels of P_CD68_/PB/N/R uptake and CAR expression were observed in TAM of orthotopic BSG mice that were locally injected with P_CD68_/PB/N/R and P_CMV_/PB/N/R (Figure S8, Supporting Information).

Induced by 10 ng ml^–1^ IL-4 previously, the RAW264.7 cells acquired M2 phenotype, and then P/PB/N/R was applied to these M2 RAW264.7 cells to gain CAR-positive RAW264.7 cells, which were selected by fluorescence-activated cell sorting (FACS) to enrich for ErbB2-CAR-macrophages (CAR-MФs). We validated the induction of the proinflammatory M1 phenotype (CD80) in CAR-MФ cells via flow cytometry, and the RP-182 peptide was likely to contribute to this result (Fig. 3E and Additional file 1: Figure S9).

The functions of the CAR-MФs were also confirmed. Cytotoxicity was tested by measuring the levels of cytoplasmic lactate dehydrogenase (LDH) released into the medium. In repetitive multiround coculture experiments with GL261-H tumor cells performed using different effector-to-target ratios, CAR-MФs showed better antitumor activity than wild RAW264.7 cells (control) in an ErbB2-CAR-mediated manner (Fig. 3F and Additional file 1: Figure S10). The results of the assessment of the antigen-specific phagocytosis of ErbB2 + beads were consistent with the cytotoxicity results (Fig. 3G, H and Additional file 1: Figure S11).

These data suggest that P_CD68_/PB/N/R can predominantly program macrophages to induce the production of M1-type CAR-MФs with stronger antitumor activity and abilities for antigen-specific phagocytosis and thus have great potential for in situ CAR editing.

### In vivo macrophage reprogramming and antitumor efficacy

**Fig. 4:**
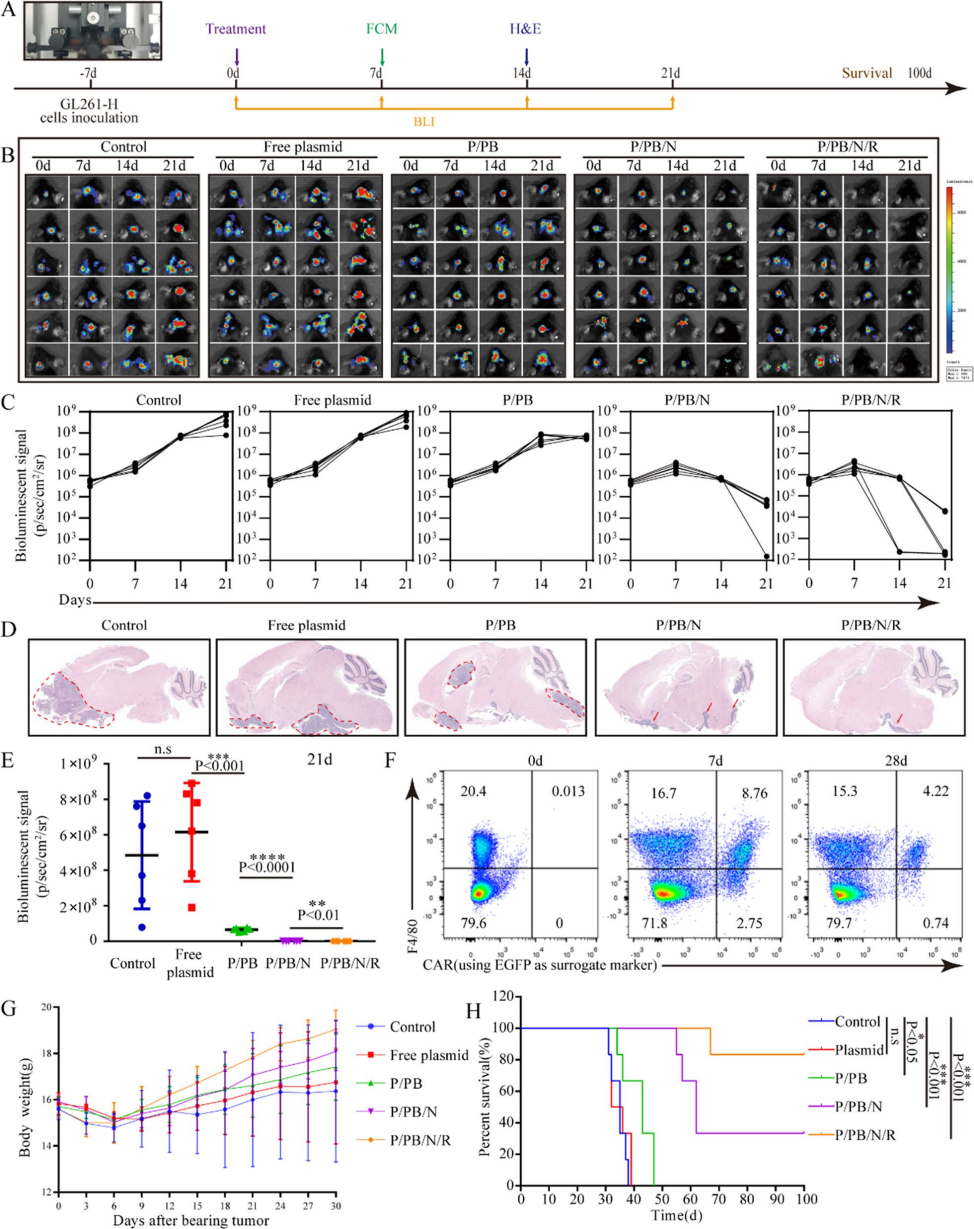
Antitumor efficacy of P/PB/N/R in an orthotopic intracranial BSG model. **A** Schematic illustration of the experimental design. **B** IVIS spectrum images and **C** quantification of the tumor signal of the mice after treatment with different nanoformulations. Each line represents one animal, and each dot reflects its whole animal photon count. **D** H&E staining of brain tissue sections obtained from mice bearing tumors. **E** In vivo bioluminescence quantified tumor signal intensity of Fig. 4B at day 21. **F** Flow cytometry of EGFP-positive cells at different time points after injection of P/PB/N/R nanoparticles. **G** Changes in body weight during the treatment period. Data are the mean ± s.d. (n = 6). **H** Survival curve of BSG mice treated with each formulation (n = 6 per group). Statistical significance was calculated via a two-tailed Student’s t test. **P < 0. 01; ***P < 0.001; ****P < 0.0001

Our goal was to selectively edit macrophages in vivo to induce the regression of cancer. Seven days postinoculation with Luci + GL261-H cells, mice were randomly grouped and treated with each nanoformulation intratumorally (Fig. 4A). Except in the control group, the dose of the nanoparticles was controlled based on the plasmid dose (2 mg/kg). Bioluminescence imaging (BLI) can be used to identify tumors early, monitor tumor growth, and efficiently measure the response to therapeutic intervention. As shown in Fig. 4B, C and Fig. 4E, the use of free plasmid did not relieve the tumor burden compared to the control group, but both P/PB and P/PB/N could relieve the tumor burden, in addition, P/PB/N/R treatment resulted in the most effective tumor regression, with no detectable tumor observed in 67% of mice. Brain tumor tissues were harvested on day 14, and tumor-bearing brain slices stained with hematoxylin and eosin were imaged. The ratios of tumor areas to brain tissue areas in the control, free plasmid-, P/PB-, P/PB/N- and P/PB/N/R-treated animals were 0.1526, 0.1517, 0.1178, 0.0684 and 0.02403, respectively (Fig. 4D and Additional file 1: Figure S12). In addition, 83.3% of P/PB/N/R-treated mice survived at least 100 days, and these mice exhibited the longest survival observed among all groups (Fig. 4H). Moreover, body weight change and histological analysis of major organs collected from mice 30 days after treatment indicated that local delivery of nanocarriers did not induce significant side effects in mice (Fig. 4G and Additional file 1: Figure S13).

To verify whether the therapeutic effect of P/PB/N/R was determined by the generation of CAR-MФs in the tumor site, P/PB/N/R was intratumorally injected 7 days postinoculation with GL261-H cells, and tumor tissues were harvested for CAR-MФ detection by flow cytometry. As shown in Fig. 4F, CAR-MФs in the tumor site were identified from day 7 to day 28. These results indicated that P/PB/N/R could edit CAR programs in tumor microenvironment in situ and bring about the regression of cancer.

### Preliminary study on the mechanism underlying nanoparticle-induced antitumor activities

**Fig. 5:**
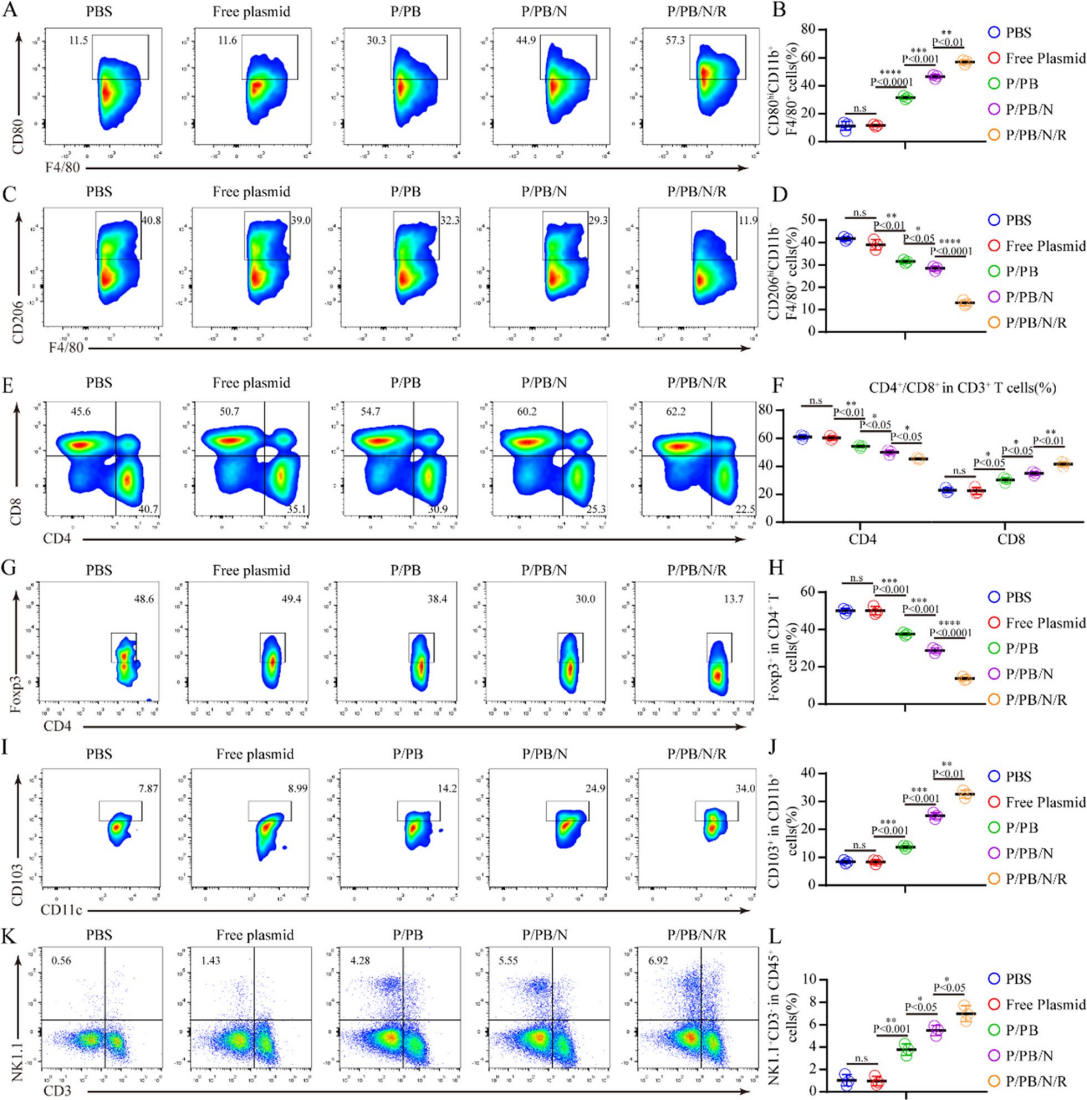
P/PB/N/R activated the adaptive immune response in tumors. **A**–**D** Representative flow cytometric analysis images and the relative quantification of M2-like macrophages (CD206hi) and M1-like macrophages (CD80hi) gating on F4/80 + CD11b + CD45 + cells. **E**–**F**) Representative flow cytometric analysis gating on CD3 + cells and absolute quantification of CD8 + and CD4 + T cells in the tumor. **G**, **H** Representative flow cytometric analysis images and relative quantification of CD4 + Foxp3 + T cells gating on CD3 + CD4 + cells. **I**, **J** Representative flow cytometric analysis images and relative quantification of CD103 + dendritic cells gating on CD45 + CD11c + cells. **K**, **L** Representative flow cytometric analysis images and relative quantification of NK1.1 + CD3- NK cells gating on CD45 + cells. Data are presented as the mean ± SEM (n = 3). Statistical significance was calculated via a two-tailed Student’s t test. *P < 0. 05; **P < 0. 01; ***P < 0.001; ****P < 0.0001

We next investigated the potent antitumor mechanism underlying the effects of the intratumoural injection of P/PB/N/R nanoparticles in vivo. Tumor-bearing mice treated with different formulations were euthanized on day 7 after inoculation, and cells isolated from harvested tumors were analyzed using flow cytometry. The proportion of M1-type macrophages in the tumor was increased after treatment with P/PB, P/PB/N and P/PB/N/R, while the proportion of M2-type macrophages showed the opposite trend (Fig. 5A–D and Additional file 1: Figure S14). Similarly, the percentage of CD8 + T cells was significantly higher in the tumors in mice subjected to P/PB/N/R treatment than that in the tumors in control mice (Fig. 5E, F and Additional file 1: Figure S15). The proliferation of CD4 + T cells was also increased (Fig. 5E, F and Additional file 1: Figure S15), while the percentages of Tregs (CD4 + Foxp3 + T cells) (Fig. 5G-H and Additional file 1: Figure S15) and myeloid-derived suppressor cells (MDSCs) were decreased (Additional file 1: Figure S16A and S17). Furthermore, an increase in the number of mature dendritic cells ( Fig. 5I, J and Additional file 1: Figure. S18) and nature killer cells (Fig. 5K, L and Additional file 1: Figure. S19) were also observed, while the number of proinflammatory neutrophils showed a decrease tendency (Additional file 1: Figure S16B and S20). The results of flow cytometry confirmed the effective innate and adaptive immune responses induced by P/PB/N/R treatment.

### In vivo* antitumor efficacy of PH/PB/N/R in the patient-derived xenograft (PDX) model*

**Fig. 6:**
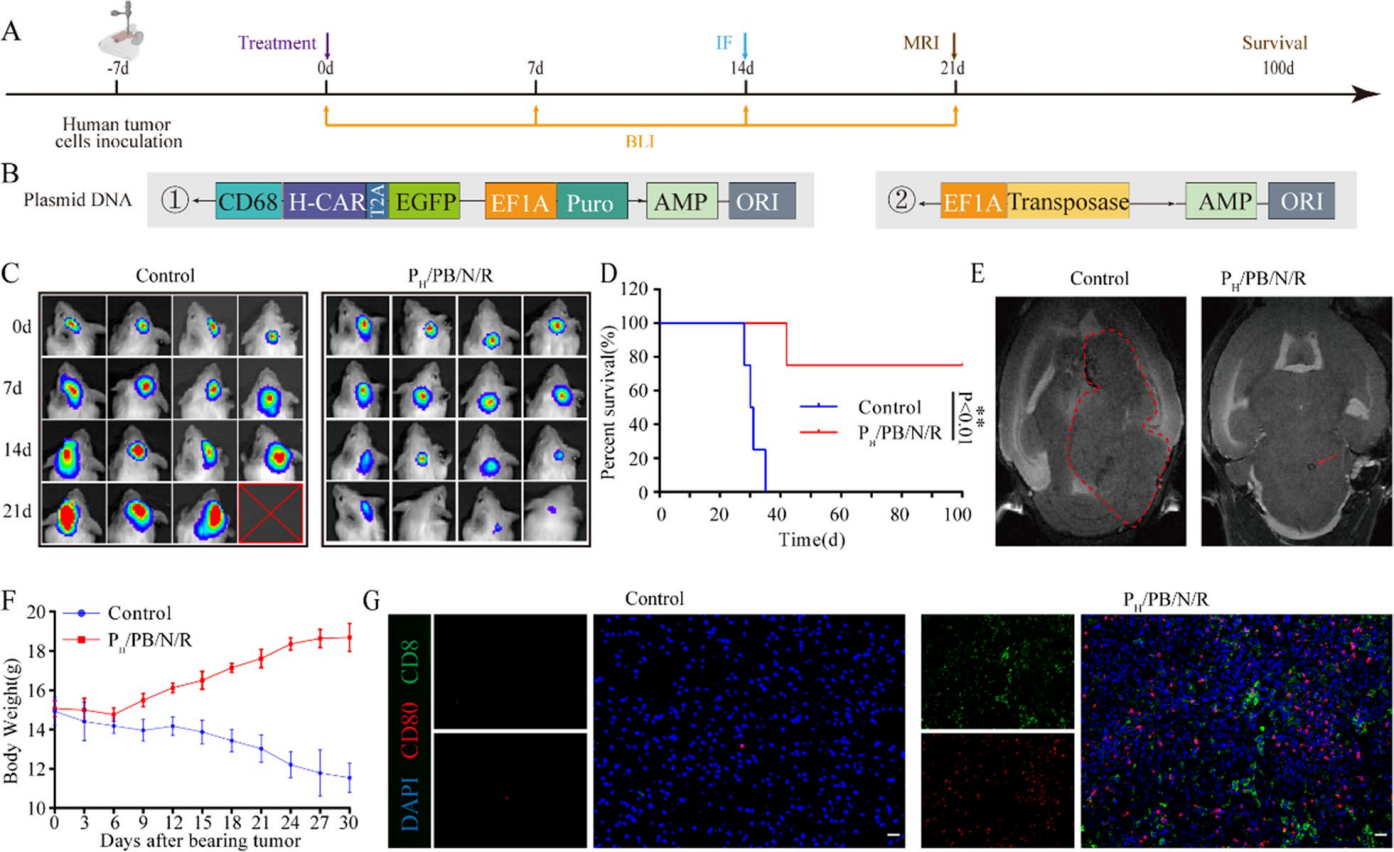
Antitumor efficacy of P_H_/PB/N/R in the PDX model. **A** Schematic illustration of the experimental design. **B** Schematic diagram of the human ErbB2-specific CAR structure. **C** In Vivo Imaging System (IVIS) spectrum images of the mice after P_H_/PB/N/R treatment on different days. **D** Survival curve of the PDX model mice treated with P_H_/PB/N/R. **E** In vivo T2-weighted MR images of tumor-bearing mice after each treatment. Both of the red circle and arrow in Fig. 6E point to the tumor site. The photos were processed with ImageJ software. **F** Weight changes in the PDX model mice treated with P_H_/PB/N/R. Data are presented as the mean ± SEM (n = 3). **G** Representative immunofluorescence images of tumors showing CD8 + T cell and CD80 + macrophage infiltration in the control and P/PB/N/R groups. Scale bars, 50 μm. Experiments were repeated three times

Encouraged by the results that were observed in immunocompetent mice, we further evaluated the antitumor efficacy against patient-derived brainstem glioma cells in a PDX model. We generated an all-human ErBb2-CAR plasmid (Fig. 6A) and targeted nanoparticles with an all-human ErBb2-CAR plasmid (P_H_/PB/N/R). An orthotopic BSG PDX model was established by intracranial injection of patient-derived brainstem glioma cells into huHSC-NOG-EXL mice (Fig. 6B and Additional file 1: Figure S21). The mice were randomly grouped before each treatment postinoculation with tumor cells. Tumor growth was then monitored with bioluminescence signals from Luc-patient-derived brainstem glioma cells (Fig. 6C and Additional file 1: Figure S22). After treatment with P_H_/PB/N/R, tumor growth in mice was significantly inhibited and 75% of the mice survived over 100 days (Fig. 6D) without abnormal body weight change (Fig. 6F). T2-weighted magnetic resonance imaging (T2W-MRI) of mouse brains bearing glioma xenografts was performed on day 21. P_H_/PB/N/R treatment exhibited superior tumor suppression activities (Fig. 6E).

Tumors were harvested and analyzed by immunofluorescence staining 7 days after treatment. Immunofluorescence staining visually indicated a marked increase in the number of macrophages and CD8 + T cells in the tumors after P_H_/PB/N/R therapy (Fig. 6G). Meanwhile, the percentage of CD8 + T and CD80hi M1-type macrophages cells was significantly higher in the tumors in mice subjected to P_H_/PB/N/R treatment than that in the tumors of control mice group (Additional file 1: Figure S23). These results are consistent with the observations in immunocompetent mice and strongly support our hypothesis that intratumoral injection of P/PB/N/R could be used to generate CAR-MФs, which would further activate the the systemic anti-tumor immune response of host.

## Conclusion

In summary, we reported a simple cancer immunotherapy strategy by injecting synthetic DNA nanocarriers into the tumor site, where intratumoral macrophages could be shaped in situ with a tumor-specific CAR to reactivate their natural role against cancerous cells. By acting as a “living” cure, the ErbB2-specific CAR-macrophages that were produced tracked and devoured cancerous cells and serially initiated innate and adaptive anti-antitumor responses to facilitate reversal of the immunosuppressive TME. Our technology therefore constitutes a practical antitumor immunotherapy for brainstem glioma and may be broadly applicable for patients suffering from other ErbB2-positive solid malignancies. Moreover, since they are stable and easy to manufacture, nanoparticles have the potential to simplify long-term storage and reduce cost. This approach might resolve several challenges facing current CAR-T-cell therapeutic approaches, including the complicated procedures associated with CAR immune cell manufacturing and the unsatisfactory clinical outcomes observed with their use in solid tumor treatment, and presents a facile solution to boost CAR technology for widespread applications.

To date, there are no strategies that can be applied therapeutically to brainstem gliomas. This system has significant implications for brainstem tumors, including inoperable cancer models such as diffuse intrinsic pontine glioma. In the Fig. 5A–D, we were pleasantly surprised to find that the groups (P/PB, P/PB/N) that did not use intact nanoformulations (P/PB/N/R) also exerted the transformation of macrophage phenotypes and we attributed it to the activated systemic anti-tumor immune response of host. In addition, one of the major regrets of this study is that the mode of administration is intratumorous administration. Although intratumoral administration is indeed a commonly means in clinical practice, considering the patient's compliance, intravenous injection reveals the distinct advantage, and great challenges remain to be conquered. Currently, we are trying to use scorpion venom peptide to modify the outer layer of the nanoparticles in order to cross the blood–brain barrier and achieve effective enrichment of the tumor site [46]. Through intravenous injection, nanoparticles can be used to reprogram macrophages in the blood. The programmed CAR-macrophages have stronger tumor-targeted migration ability. It has also been reported that M1 macrophages, overexpression of integrin α4, β1, and macrophage-1 antigen (Mac-1) on the surface, also allows them to penetrate the blood–brain barrier and effectively target brain tumors. Therefore, this therapy might be effective for targeting the macrophages in the tumor microenvironment if delivered intravenously. In addition, the therapeutic effect of humanized mice is not as bright as that of immunocompetent mice, and a possible cause may be that the immune system of humanized mice is not sound enough. This work revealed a cost-effective and safe treatment method of BSG, which are highly valuable in guiding the future research and development of clinical therapy of aggressive cancers.

## Materials and methods

### Cell lines

BMDMs were harvested from mouse femurs to prepare mouse macrophages. These cells were then cultured in Dulbecco's Modified Eagle’s Medium (DMEM; containing 10% fetal bovine serum (FBS), penicillin/streptomycin, glutamine, and HEPES) and treated with 20 ng ml^−1^ granulocyte–macrophage colony-stimulating factor (GM-CSF) for 7 days. Next, they were conditioned with M2 macrophage-conditioned medium (20 ng ml^−1^ IL-4). Both the murine GL261 and Luci + GL261 cell lines (gifted from Jiangsu University) were transfected with 10 μg plasmids that encoded cDNAs for ErbB2 (OriGene Technologies, RC400096) to obtain the ErbB2 + and Luci + GL261 ErbB2 cell lines (named GL261-H and Luci + GL261-H cell lines), and the Luci + GL261-H cell line was used for in vivo bioluminescent imaging. The RAW264.7 cell line and the 293 T cell line (American Type Culture Collection) were cultured in complete DMEM with 10% heat-inactivated FBS, 100 U ml^–1^ penicillin and 100 μg ml^–1^ streptomycin. Human hematopoietic stem cells were kindly donated by the YINFENG Biological Group, LTD.

### Animals

C57BL/6 mice (4 w, ♀) were purchased from Huafukang Biotech. All animal experiments were approved by and performed in compliance with the guidelines and regulations of the Institutional Animal Care and Use Committee of the Cheeloo College of Medicine, Shandong University (21074). Animals were housed in a controlled environment, with the following target conditions: temperature, 19–25 °C; relative humidity, 30–60%. An electronic time-controlled lighting system was used to provide a 12 h light/12 h dark cycle.

### Plasmid generation

In this study, piggyBac transposon gene expression vectors were used, which contained the CD8α hinge and transmembrane domains and the CD3ζ endo domain. The ErbB2-specific scFv domain used to generate ErbB2-CAR. This CAR (named CD68 plasmid) construct with reporter proteins was generated by fusing GFP with the ErbB2-specific scFv domain, which was custom-cloned by TsingKe and separated by a T2A sequence. The only difference between this and the CMV plasmid was the use of the CMV promoter instead of the CD68 promoter.

### C32-122–NLS peptide conjugation

A solution of 12 mg 4-(maleinimido) phenyl isocyanate (PMPI) in DMSO (20 mg ml^–1^) was added to 86 mg C32-122 polymer in DMSO (100 mg ml^–1^). The solution was mixed at room temperature (RT) for 3 h. The C32-122-maleimide derivative was added to a solution of 100 mg NLS peptide in 5.3 ml DMSO containing tris(2-carboxyethyl) phosphine hydrochloride (TCEP·HCl; 3 mg ml^–1^). The solution was mixed at RT for 3 h and then filtered through a 7 k Zeba spin column equilibrated with DMSO. The DMSO was evaporated under vacuum overnight. The C32-122-peptide conjugate was redissolved in DMSO to 100 mg ml^–1^ C32-122 and stored at − 20 °C.

### Peptide synthesis

The NLS sequence (Cys-Arg-DArg-Arg-Lys-NH2) and RP-182 peptide (KFRKAFKRFF) were custom synthesized by AnaSpec Inc. A cysteine was added to the N-terminus of the peptide for linkage to the C32-122 polymer and the PGA.

### PGA–RP-182 peptide conjugation

PGA was dissolved in water to achieve a concentration of 20 mg ml^–1^ and then sonicated for 10 min in a bath sonicator. An equal volume of ethyl-N′-(3-dimethylaminopropyl) carbodiimide·HCl in water (4 mg ml^–1^, 16 equiv.) was added, and the solution was mixed at RT for 5 min. The resulting activated PGA was added to a solution of RP-182 peptide in PBS at a 4:1 molar ratio and mixed at RT for 6 h. Excess reagents were removed by dialysis (1000 MWCO Slide-A-Lyzer Dialysis Cassette) against PBS for 24 h, followed by filtration through a 40 k Zeba spin column. The peptide concentration was determined using a NanoDrop 2000 Spectrophotometer (Thermo Scientific).

### Nanoparticle preparation

All components were diluted in sodium acetate buffer (25 mM, pH 5.2) to the following concentrations: DNA, 0.1 mg ml^–1^; C32-122 and C32-122–NLS, 3.14 mg ml^–1^; PGA–peptide, 0.45 mg ml^–1^ peptide. To prepare the particles, C32-122–NLS was added to DNA at a PBAE:DNA mass ratio of 15:1. The mixture was vortexed gently for 10 s and then incubated at RT for 10 min. Unconjugated C32-122 was then added to the complex at a PBAE:DNA mass ratio of 15:1. The mixture was vortexed gently for 10 s and then incubated at RT for 5 min. PGA peptide was then added at a peptide:DNA mass ratio of 2.5:1. The mixture was vortexed gently for 10 s and then incubated at RT for 10 min.

### Nanoparticle characterization

The diameters and zeta potentials of the nanoparticles were determined by a dynamic light scattering (DLS) method using a Malvern Zetasizer Nano ZS90 (Worcestershire, UK). The morphology of the nanoparticles was examined under transmission electron microscopy (JEM-1200EX, JEOL). The diluted sample solution was dropped onto a copper grid followed by negative staining with phosphotungstic acid (2 wt%, pH adjusted to 6.5 with sodium hydroxide), dried for 20 min and then observed under TEM.

In vitro cellular uptake of FAM-P/PB/N/R nanocarriers (using FAM-modified plasmid instead of the plasmid used to generate P/PB/N/R nanocarriers) by Different Cell Types.

The cellular uptake of the P/PB/N/R nanocarriers by different cell types (BMDMs and the RAW264.7, 293 T and GL261-H cell lines) was analyzed. The FAM plasmid was encapsulated into the FAM-P/PB/N/R nanocarriers (FAM-P/PB/N/R nanocarriers) to facilitate detection. For flow cytometry analysis, BMDMs or RAW264.7, 293 T and GL261-H cell lines (2 × 10^5^ cells/well) were seeded in six-well plates for 24 h. Then, the cells were incubated with FAM-P/PB/N/R nanocarriers at a plasmid dose of 50 nM. After incubation for 6 h, the cells were washed three times with cold PBS, trypsinized, and collected for FCM analyses.

For confocal laser scanning microscopy (CLSM) observation, BMDMs or RAW264.7, 293 T and GL261-H cell lines (5 × 10^4^ cells/well) were seeded onto coverslips in 24-well plates for 24 h. After incubation for 6 h, the cells were treated with the formulations described above, and then the cytoskeletons and nuclei were counterstained with rhodamine B(red) and DAPI (blue), respectively, according to the standard protocols provided by the suppliers. Then, the cellular uptake behavior was visualized under CLSM (CLSM 710, Carl Zeiss Inc., Jena, Germany).

### In vitro* transfection of RAW264.7 cells with synthetic DNA nanocarriers*

The CD68 plasmid, which simultaneously expresses EGFP under the control of the CD68 promoter, was used to determine the plasmid transfection efficiency of the synthetic DNA nanocarriers, and the CMV plasmid with the CMV promoter was used as a control. Briefly, RAW264.7 cells (2 × 10^5^ cells/well) were seeded in six-well plates for 24 h, and the cells were then incubated with synthetic DNA nanocarriers with different structural integrities (including free plasmid, P/PB nanocarriers containing plasmid DNA and PBAE, P/PB/N nanocarriers containing plasmid DNA and PBAE C32-122-NLS, P/PB/N/R nanocarriers containing plasmid DNA, PBAE C32-122-NLS and PGA–peptide) at plasmid doses of 50 nM. After incubation for 6 h, the medium was replaced with fresh medium, and the cells were further incubated for 48 h. Afterwards, the percentage of EGFP-positive cells was analyzed by a flow cytometry assay (FACS Calibur flow cytometer, BD Biosciences, USA), and after further incubation for 48 h, EGFP expression was analyzed by western blotting. A monoclonal antibody against EGFP (Abcam, cat. no. ab184601) was used at a dilution of 1:1000, and goat anti-mouse IgG-HRP (1:5000, Abcam, cat. no. ab6789) was used as the secondary antibody. The results were visualized using an ImageQuant LAS 4000 mini system (GE Healthcare, UK).

### In vitro* transfection of different cells with P/PB/N/R*

The uptake of the P/PB/N/R nanoparticles by different cell types (BMDMs and the RAW264.7, 293 T and GL261-H cell lines) was analyzed. FAM-labeled DNA was encapsulated into the P/PB/N/R nanoparticles to facilitate detection. For flow cytometry analysis, BMDMs or RAW264.7, 293 T or GL261-H cells (2 × 10^5^ cells/well) were seeded in six-well plates for 24 h. Then, the cells were incubated with nanoparticles loaded with FAM-labeled DNA at a plasmid DNA dose of 50 nM. After incubation for 6 h, the cells were washed three times with cold PBS, trypsinized, and collected for FCM analyses.

For CAR expression detection, BMDMs or RAW264.7, 293 T or GL261-H cells (2 × 10^5^ cells/well) were seeded in six-well plates for 72 h. After incubation with P/PB/N/R nanocarriers loaded with CD68 plasmid or CMV plasmid for 6 h, the medium was replaced with fresh medium, and the cells were further incubated for 48 h. Subsequently, the percentage of EGFP-positive cells was analyzed by a flow cytometry assay (Beckman Coulter GALLIOS Cytometer, CA, US), and EGFP positive cells were named CAR-MФs.

### Macrophage phenotype determination and cell sorting

All flow cytometry antibodies were purchased from Biolegend. Cells were acquired by flow cytometry (Beckman Coulter GALLIOS Cytometer, CA, US). For experiments involving fluorescence-activated cell sorting, a FACSMelody cell sorter (BD Biosciences, US) was used to obtain CAR-RAW264.7 cells via GFP.

### Cytotoxicity

GL261-H cells were seeded in 96-well plates and grown for 16 h. CAR-MФs and RAW264.7 cells were then added to the wells at different ratios of 10:1, 5:1, 2.5:1 and 1.25:1 for 6 h. The release of LDH into the medium was quantified using a Cytotox96 nonradioactive cytotoxicity kit (Promega, Madison, WI, USA) following the manufacturer’s instructions. The amount of LDH released (% cytotoxicity) was calculated according to the formula:

$${\text{Cytotoxicity }}\left( \% \right)\,\, = \,\left( {{\text{OD49}}0{\text{ for experimental release}}\, - \,{\text{OD49}}0{\text{ for spontaneous release}}} \right)/\left( {{\text{OD49}}0{\text{ for maximum release}}\, - \,{\text{OD49}}0{\text{ for spontaneous release}}} \right)\, \times \,{1}00.$$

The spontaneous release was the amount of LDH released from the cytoplasm of GL261-H cells, whereas the maximum release was the amount of LDH released by total lysis of cells in the experimental group. The assay was replicated three times.

### Phagocytosis assay

The cy5-labeled ErbB2 protein was linked with glutathione-coated polystyrene particles (4.0–4.9 µm diameter, Spherotech) and then resuspended in PBS for use in phagocytosis assay experiments. The functionalized beads were incubated with macrophages at a ratio of 1:1 for 45 min, and then the cells were imaged through high-speed confocal microscopy (Andor Dragonfly 200, UK).

### Toxicity studies

To measure the potential in vivo toxicities of macrophage-targeted DNA nanocarriers, we injected mice intravenously with various formulations. Animals were then euthanized with carbon dioxide to retrieve organs, which were washed with PBS before fixation in 4% paraformaldehyde. The tissues were processed routinely, and sections were stained with hematoxylin and eosin.

### Immunofluorescence staining

Tumors were collected from the mice and snap-frozen in optimal cutting temperature medium. Tumor sections were cut using a cryotome, mounted on slides and stained with different primary antibodies, including CD8 (Abcam, cat. no. ab22378) and CD80 (Abcam, cat. no. ab100790), overnight at 4 °C following the manufacturer’s instructions. Following the addition of fluorescently labeled secondary antibodies (goat anti-rat IgG (H + L; Thermo Fisher Scientific, cat. no. A18866) and goat anti-rabbit IgG (H + L; Thermo Fisher Scientific, Cat. no. A32733)), the slides were analyzed with high-speed confocal microscopy (Andor Dragonfly 200, UK). All antibodies used in the experiments were diluted 200 times.

### Western blotting

Equal amounts of protein (measured using a bicinchoninic acid protein assay kit, BCA) were mixed with an equal volume of 2 × Loading buffer and boiled at 95 °C for 5 min. After gel electrophoresis and protein transformation, anti-ErbB2 antibody at a dilution of 1:1,000 (Abcam, Cat. no. ab134182) and anti-GAPDH antibody (Abcam, Cat. no. ab9485) at a 1:5,000 dilution were used as primary antibodies. The secondary antibody used for these blots was a goat anti-mouse antibody (Abcam, Cat. no. ab6721).

### In vivo* tumour models and treatment*

Briefly, 5 × 10^5^ Luc + GL261-H cells were resuspended in 8 μl of cold PBS and stereotactically implanted into the pons of a mouse (1 mm right to and 3 mm posterior to lambda, and 3.5 mm depth from the skull). After loading the injection into the animal, the skin was glued to close the incision. Free plasmid and various nanoformulations were intratumourally injected into GL261-H-bearing mice at a plasmid dose of 2 mg kg^−1^ body weight on day 7, and the PBS group underwent the same way of injection with equal volume of PBS simultaneously. Meanwhile, the tumour tissues were collected on days 0 and 3 for flow cytometry analysis on immune cells (T cells, B cells and macrophages) stained with antibodies to verify the percentage of FAM-positive cells and EGFP-positive cells, respectively. In addition, the tumour tissues were also collected on days 0, 7, and 28 for flow cytometry analysis to quantify EGFP expression in macrophages. The tumour volumes were determined via in vivo non-invasive optical biophotonic imaging (PerkinElmer IVIS Spectrum) every 7 days. The mice’s body weights and survival rates were monitored simultaneously. Furthermore, on day 14, the tumour tissues were harvested and fixed in formalin and embedded in paraffin, and 10 μm sections were cut to reveal coronal views of the brains. The slices were processed for standard H&E staining and then were counterstained with DAPI, and the tumour area was calculated by using ImageJ software.

Luc + PDG cells were injected stereotactically into the right striatum of huHSC-NOG-EXL female mice. PBS and P/PB/N/R was administered intratumourally into huHSC-NOG-EXL tumour bearing mice 7 days. The tumour size was monitored every week through BLI.

### Flow cytometry

Tumors collected from mice were divided into small pieces and homogenized in cold staining buffer to form single-cell suspensions in the presence of digestive enzyme. Cells were stained with fluorescence-labeled antibodies against CD45 (Biolegend, Cat. no. 103116, clone 30-F11), CD3 (Biolegend, Cat. no. 100217, clone 17A2), CD4 (Biolegend, Cat. no. 100429, clone GK1.5), CD8a (Biolegend, Cat. no. 100721, clone 53–6.7), Foxp3 (Biolegend, cat. no. 126404, clone MF-14), NK-1.1 (Biolegend, Cat. no. 108709, clone PK136), CD62 L (Biolegend, Cat. no. 104407, clone MEL-14), CD44 (Biolegend, Cat. no. 103005, clone IM7), CD11b (Biolegend, Cat. no. 101205, clone M1/70), CD206 (Biolegend, Cat. no. 141731,clone C068C2), CD80 (Biolegend, Cat. no. 104733, clone 16-10A1), F4/80 (Biolegend, Cat. no. 123131, clone BM8), Ly-6G (Biolegend, Cat. no. 127615, clone 1A8), Gr-1 (Biolegend, Cat. no. 108407, clone RB6-8C5), CD86 (Biolegend, Cat. no. 105011, clone GL-1), CD103 (Biolegend, Cat. no. 121415, clone 2E7), and CD11c (Biolegend, Cat. no. 117307, clone N418), and were treated with Fixable Viability Dye (Biolegend, cat. no. 423101), Cell Stimulation Cocktail (Biolegend, cat. no. 423303), Intracellular Staining Perm Wash Buffer (10X) (Biolegend, Cat. no. 421002), and Fixation Buffer (Biolegend, Cat. no. 420801).

### MRI

At the endpoints of various treatments, the mice were subjected to in vivo T2-weighted MRI studies by using the following parameters: repetition time (TR)/echo time (TE) = 2500/35 ms; acquisition matrix = 256 × 256; field of view = 30 × 30 mm; number of slices = 22; slice thickness = 0.4 mm, Rapid Imaging with Refocused Echoes (RARE) factor = 8.

### Human tumor specimens

Patient tumor sections were obtained from the Department of Neurosurgery at Shandong Provincial Hospital Affiliated with Shandong First Medical University. All the samples were obtained with written informed consent and collected using a standard protocol approved under the Review Board of Shandong First Medical University (Approval no. 2020-272). The sections were incubated with primary antibodies against ErbB2 (dilution of 1:1000; Abcam, cat. no. ab134182).

### Statistical analysis

Each figure legend denotes the statistical test used. Unless otherwise indicated, all data are shown as the means ± standard deviations (SD). Statistical analysis was performed by one-way analysis of variance (ANOVA) in GraphPad Prism 7.0 (GraphPad). For all figures, significant differences between groups are indicated by * P < 0.05, ** P < 0.01, *** P < 0.001 and **** P < 0.0001. Results with p values of < 0.05 were considered statistically significant in all analyses (95% confidence level).

## Supplementary Information

**Additional file 1: Figure S1.** A) Immunohistochemical examination of intratumoural and peritumoural expression of ErbB2. B) The expression of ErbB2 in tumor and normal tissues analyzed by Western blot. C) Expression of ErbB2 in the GL261-H cells (ErbB2+) via flow cytometry and D) Western blot. **Figure S2.** A) Map of piggybac-CMV promoter-ErbB2-CAR and B) part of the sequencing results for anti-mouse ErbB2 ScFv in the CAR plasmid. **Figure S3.** A) Map of piggybac-CD68 promoter-ErbB2-CAR and B) part of the sequencing results for the CD68 promoter in the plasmid. **Figure S4.** A) Map of the piggybac-CD68 promoter-human ErbB2-CAR and B) part of the sequencing results for anti-human ErbB2 ScFv in the CAR plasmid. **Figure S5.** Stability of P/PB/N/R in 50% serum, as evaluated by determining changes in the nanocomplex size by DLS over 7 days. Data represent mean ± SEM (n = 3). **Figure S6.** Confocal images of RAW264.7 cells treated with PBS and different nanoparticles containing the FAM plasmid (green). The cytoskeletons and nuclei were counterstained with RhB (red) and DAPI (blue), respectively (scale bar, 5 μm). **Figure S7.** The quantitative analysis of western blot in Figure 3D. Data are shown as the mean ± SEM (n = 3). **Figure S8.** Percentage of FAM-positive and CAR-positive T cells, B cells and macrophages in tumour after locally injection of P_CD68_/PB/N/R and P_CMV_/PB/N/R nanocarriers. Data are shown as the means ± SEM (n = 3). **Figure S9. **Flow cytometry analysis of the M1-related marker CD80 in RAW264.7 transfected with nanoparticles with a sham plasmid (left) and CAR-MФ (right) cells. **Figure S10. **LDH cytotoxicity assay of RAW264.7 transfected with nanoparticles with a sham plasmid and CAR-MФs. Cytotoxicity was evaluated at different effector:target cell ratios in GL261-H cells. Data are representative of three independent experiments. Statistical significance was calculated via a two-tailed Student’s t test. **P < 0. 01. **Figure S11. **Confocal images (A) and statistical graph of phagocytotic ability (B) of RAW264.7 transfected with nanoparticles with a sham plasmid and CAR-MФ for the uptake of ErbB2 beads. Images from left to right show DAPI-stained cell nuclei (blue), CFSE-stained cells (green), ErbB2 beads (red), and merged images. Data are shown as the mean ± SEM (n = 3). Statistical significance was calculated via a two-tailed Student’s t test. **P < 0. 01. **Figure S12.** Quantitative analysis of the ratio of tumor size to the size of the brain. Data are presented as the mean ± SEM (n = 3); statistical significance was calculated via two-tailed Student’s t test. *P < 0. 05. **Figure S13.** H&E staining of major organs from mice treated with different formulations (scale bar, 100 μm). **Figure S14.** Gating strategies for M1 and M2 macrophages in BSG tumor models. **Figure S15.** Gating strategies for CD8+ T cells, CD4+ T cells and Tregs in BSG tumor models. **Figure S16.** A) Representative flow cytometric analysis images (left) and relative quantification (right) of MDSCs (CD11b+Gr-1+) gating on CD45+ cells. Data are presented as the mean ± s.e.m. (n = 3). B) Representative flow cytometry analysis images and relative quantification of CD11b+ly6G+ neutrophil cells gating on CD45+ cells. Data are presented as the mean ± SEM (n = 3). Statistical significance was calculated via two-tailed Student’s t test. *P < 0.05; **P < 0.01. **Figure S17.** Gating strategies for MDSCs in BSG tumor models. **Figure S18.** Gating strategies for mature dendritic cells in BSG tumor models. **Figure S19.** Gating strategies for nature killer cells in BSG tumor models. **Figure S20.** Gating strategies for neutrophils in BSG tumor models. **Figure S21.** A) An image of the human luc+ EGFP+ brainstem cancer cell line in bright field and B) an image obtained under fluorescence microscope. **Figure S22. **The quantitative fluorescence intensity of IVIS spectrum images in Figure 6C. **Figure S23. **Relative quantification of CD80hiF4/80+CD11b+ cells and CD8+CD3+ cells gating on CD45+ cells. Data are presented as the mean ± SEM (n = 3). Statistical significance was calculated via a two-tailed Student’s t test. ****P < 0.0001.

## Data Availability

Data sharing availability to this article.
