# Supplementary material for: Convection-enhanced delivery of nanoencapsulated gene locoregionally yielding ErbB2/Her2-specific CAR-macrophages for brainstem glioma immunotherapy
Source: J Nanobiotechnology. 2023 Feb 20;21:56. doi: 10.1186/s12951-023-01810-9 (PMC9940362; doi:10.1186/s12951-023-01810-9)
Supplement: Supplementary file 1 — Additional file 1: Figure S1. A) Immunohistochemical examination of intratumoural and peritumoural expression of ErbB2. B) The expression of ErbB2 in tumor and normal tissues analyzed by Western blot. C) Expression of ErbB2 in the GL261-H cells (ErbB2+) via flow cytometry and D) Western blot. Figure S2. A) Map of piggybac-CMV promoter-ErbB2-CAR and B) part of the sequencing results for anti-mouse ErbB2 ScFv in the CAR plasmid. Figure S3. A) Map of piggybac-CD68 promoter-ErbB2-CAR and B) part of the sequencing results for the CD68 promoter in the plasmid. Figure S4. A) Map of the piggybac-CD68 promoter-human ErbB2-CAR and B) part of the sequencing results for anti-human ErbB2 ScFv in the CAR plasmid. Figure S5. Stability of P/PB/N/R in 50% serum, as evaluated by determining changes in the nanocomplex size by DLS over 7 days. Data represent mean ± SEM (n = 3). Figure S6. Confocal images of RAW264.7 cells treated with PBS and different nanoparticles containing the FAM plasmid (green). The cytoskeletons and nuclei were counterstained with RhB (red) and DAPI (blue), respectively (scale bar, 5 μm). Figure S7. The quantitative analysis of western blot in Figure 3D. Data are shown as the mean ± SEM (n = 3). Figure S8. Percentage of FAM-positive and CAR-positive T cells, B cells and macrophages in tumour after locally injection of PCD68/PB/N/R and PCMV/PB/N/R nanocarriers. Data are shown as the means ± SEM (n = 3). Figure S9. Flow cytometry analysis of the M1-related marker CD80 in RAW264.7 transfected with nanoparticles with a sham plasmid (left) and CAR-MФ (right) cells. Figure S10. LDH cytotoxicity assay of RAW264.7 transfected with nanoparticles with a sham plasmid and CAR-MФs. Cytotoxicity was evaluated at different effector:target cell ratios in GL261-H cells. Data are representative of three independent experiments. Statistical significance was calculated via a two-tailed Student’s t test. **P < 0. 01. Figure S11. Confocal images (A) and statistical graph of phagocyto [file 12951_2023_1810_MOESM1_ESM.docx]

**Convection-enhanced Delivery of** **Nanoencapsulated Gene Locoregionally Yielding** **ErbB2/Her2-specific CAR-macrophages for Brainstem Glioma Immunotherapy**

Lin Gao^1#^, Chongdeng Shi^1#^, Zhenmei Yang^1^, Weiqiang Jing^2^, Maosen Han^1^, Jing Zhang^1^, Cai Zhang^1^, Chunwei Tang^1^, Yuanmin Dong^1^, Ying Liu^1^, Chen Chen^1*^, Xinyi Jiang^1*^

^1^NMPA Key Laboratory for Technology Research and Evaluation of Drug Products and Key Laboratory of Chemical Biology (Ministry of Education), School of Pharmaceutical Sciences, Cheeloo College of Medicine, Shandong University, 44 Cultural West Road, Shandong Province, 250012, China

^2^Department of Urology, Qilu Hospital, Cheeloo College of Medicine, Shandong University, 107 Cultural West Road, Shandong Province, 250012, China

^#^These authors contributed equally.

^*^Corresponding author: Chen Chen, Xinyi Jiang.

Phone/Fax：+86-15662758621

Email：[xinyijiang@sdu.edu.cn](mailto:xinyijiang@sdu.edu.cn); arielchen910607@gmail.com

**Additional file**


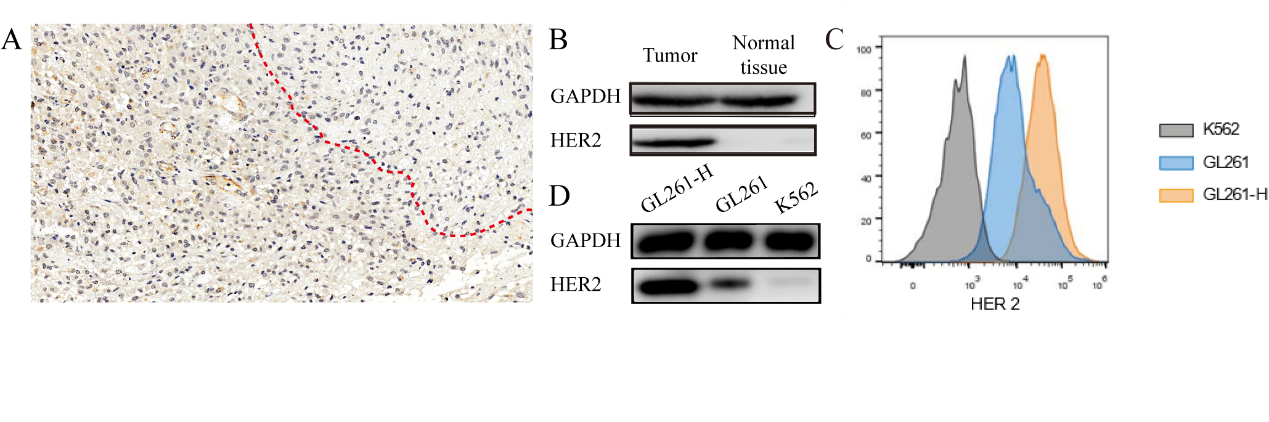


**Figure S1.** A) Immunohistochemical examination of intratumoural and peritumoural expression of ErbB2. B) The expression of ErbB2 in tumor and normal tissues analyzed by Western blot. C) Expression of ErbB2 in the GL261-H cells (ErbB2+) via flow cytometry and D) Western blot.


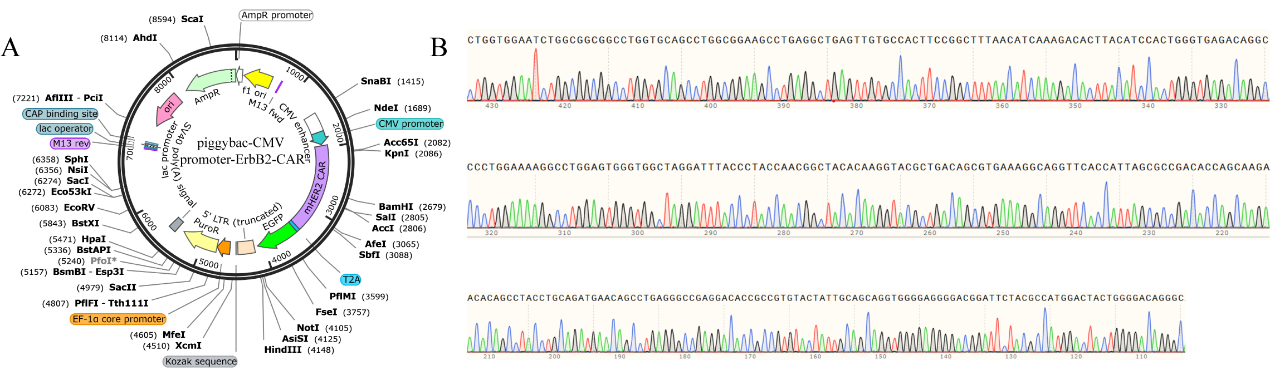


**Figure S2.** A) Map of piggybac-CMV promoter-ErbB2-CAR and B) part of the sequencing results for anti-mouse ErbB2 ScFv in the CAR plasmid.


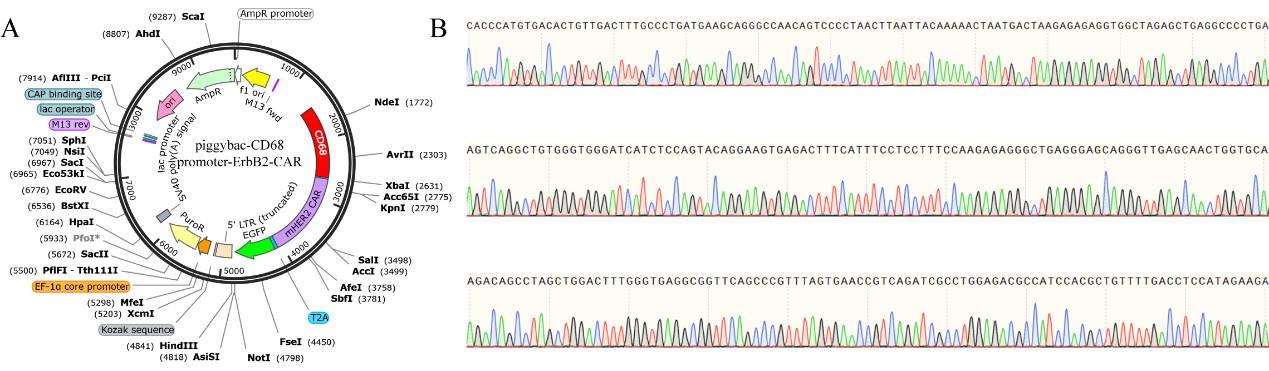


**Figure S3.** A) Map of piggybac-CD68 promoter-ErbB2-CAR and B) part of the sequencing results for the CD68 promoter in the plasmid.


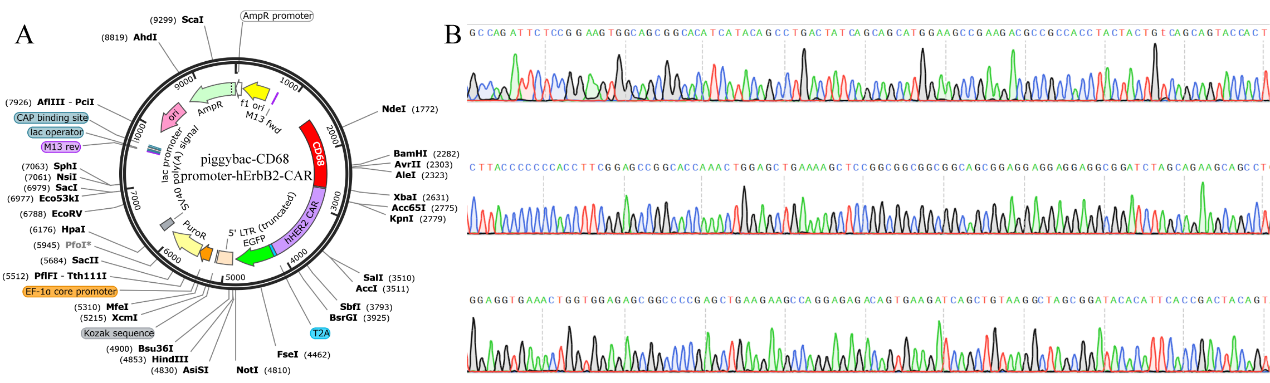


**Figure S4.** A) Map of the piggybac-CD68 promoter-human ErbB2-CAR and B) part of the sequencing results for anti-human ErbB2 ScFv in the CAR plasmid.


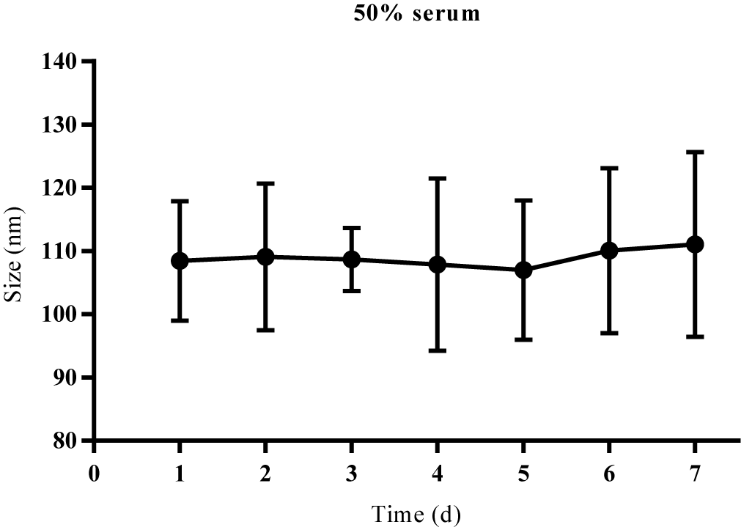


**Figure S5.** Stability of P/PB/N/R in 50% serum, as evaluated by determining changes in the nanocomplex size by DLS over 7 days. Data represent mean ± SEM (n = 3).


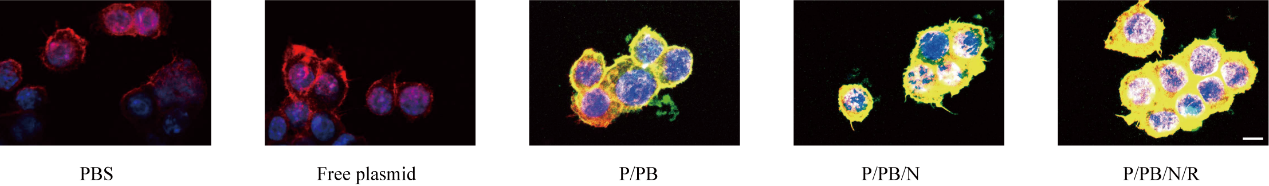


**Figure S6.** Confocal images of RAW264.7 cells treated with PBS and different nanoparticles containing the FAM plasmid (green). The cytoskeletons and nuclei were counterstained with RhB (red) and DAPI (blue), respectively (scale bar, 5 μm).


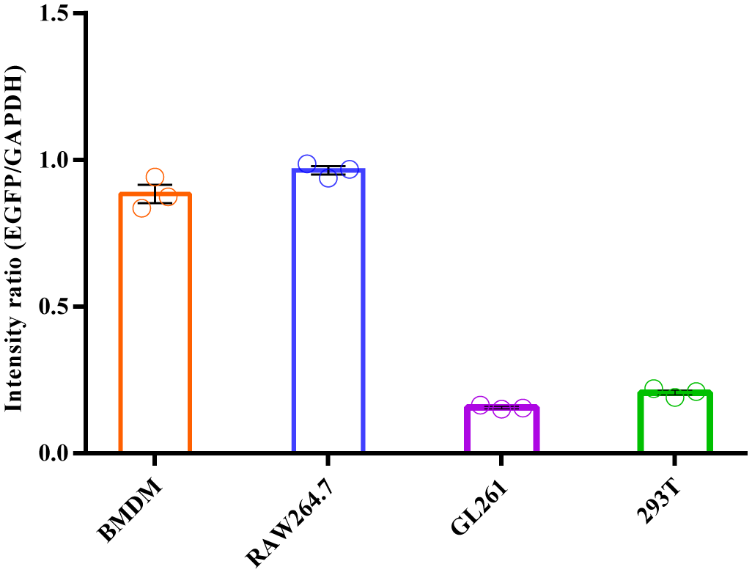


**Figure S7.** The quantitative analysis of western blot in Figure 3D. Data are shown as the mean ± SEM (n = 3).


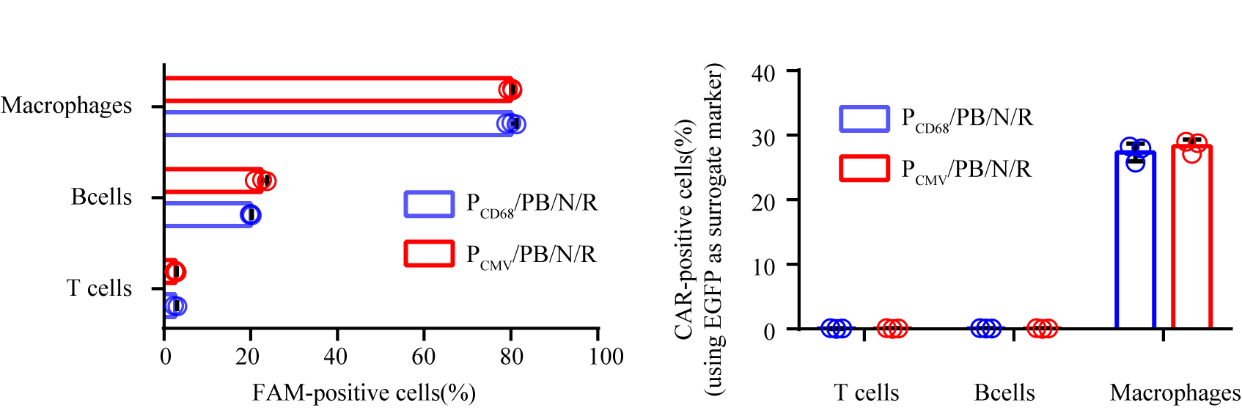


**Figure S8.** Percentage of FAM-positive and CAR-positive T cells, B cells and macrophages in tumour after locally injection of P_CD68_/PB/N/R and P_CMV_/PB/N/R nanocarriers. Data are shown as the means ± SEM (n = 3).
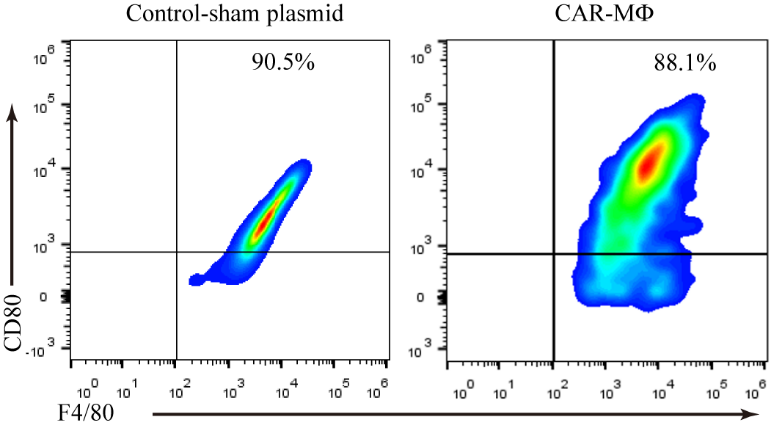


**Figure S9.** Flow cytometry analysis of the M1-related marker CD80 in RAW264.7 transfected with nanoparticles with a sham plasmid (left) and CAR-MФ (right) cells.


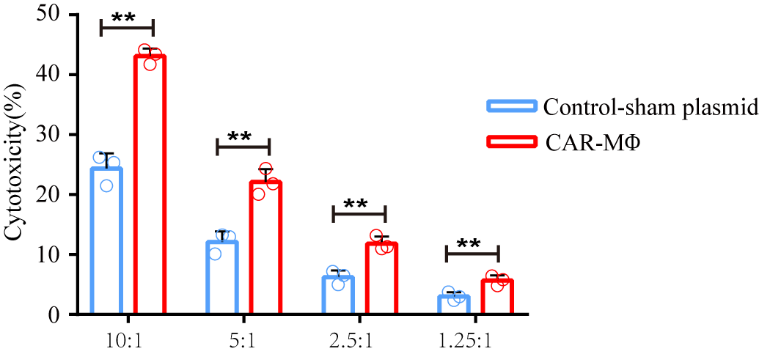


**Figure S10.** LDH cytotoxicity assay of RAW264.7 transfected with nanoparticles with a sham plasmid and CAR-MФs. Cytotoxicity was evaluated at different effector:target cell ratios in GL261-H cells. Data are representative of three independent experiments. Statistical significance was calculated via a two-tailed Student’s t test. **P < 0. 01.


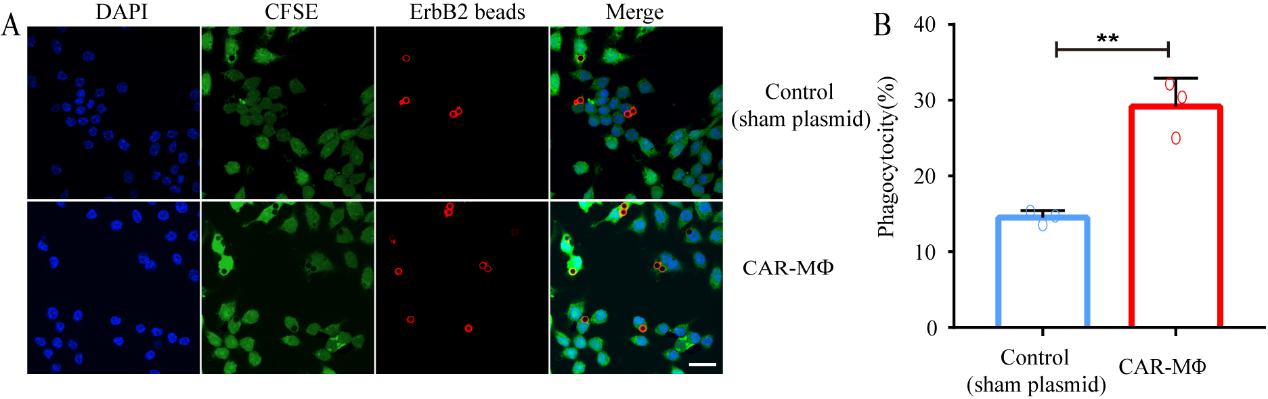


**Figure S11.** Confocal images (A) and statistical graph of phagocytotic ability (B) of RAW264.7 transfected with nanoparticles with a sham plasmid and CAR-MФ for the uptake of ErbB2 beads. Images from left to right show DAPI-stained cell nuclei (blue), CFSE-stained cells (green), ErbB2 beads (red), and merged images. Data are shown as the mean ± SEM (n = 3). Statistical significance was calculated via a two-tailed Student’s t test. **P < 0. 01.


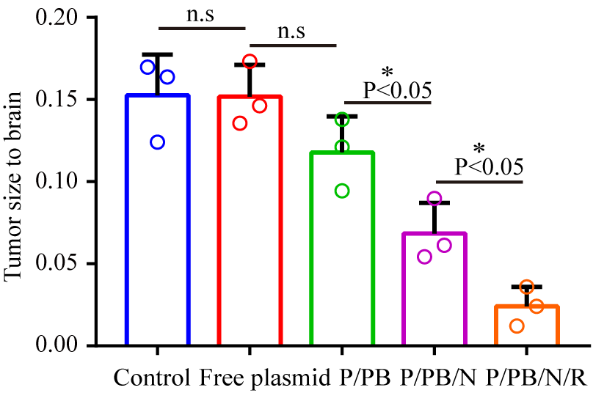


**Figure S12.** Quantitative analysis of the ratio of tumor size to the size of the brain. Data are presented as the mean ± SEM (n = 3); statistical significance was calculated via two-tailed Student’s t test. *P < 0. 05.


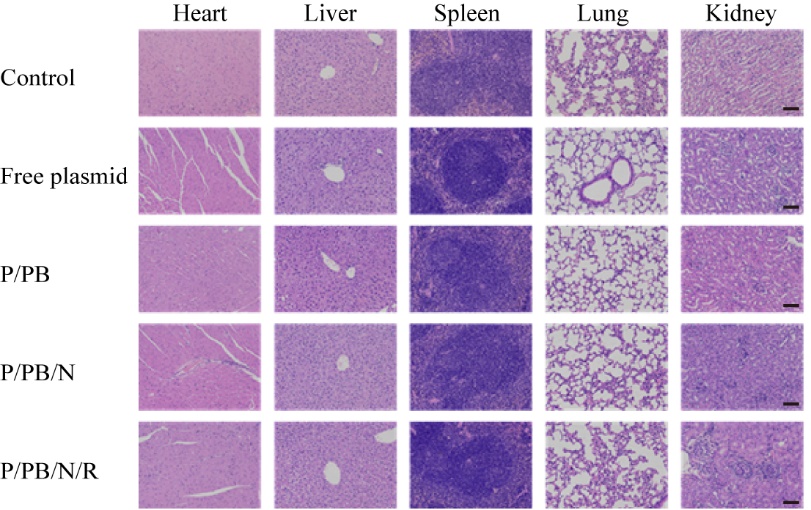


**Figure S13.** H&E staining of major organs from mice treated with different formulations (scale bar, 100 μm).


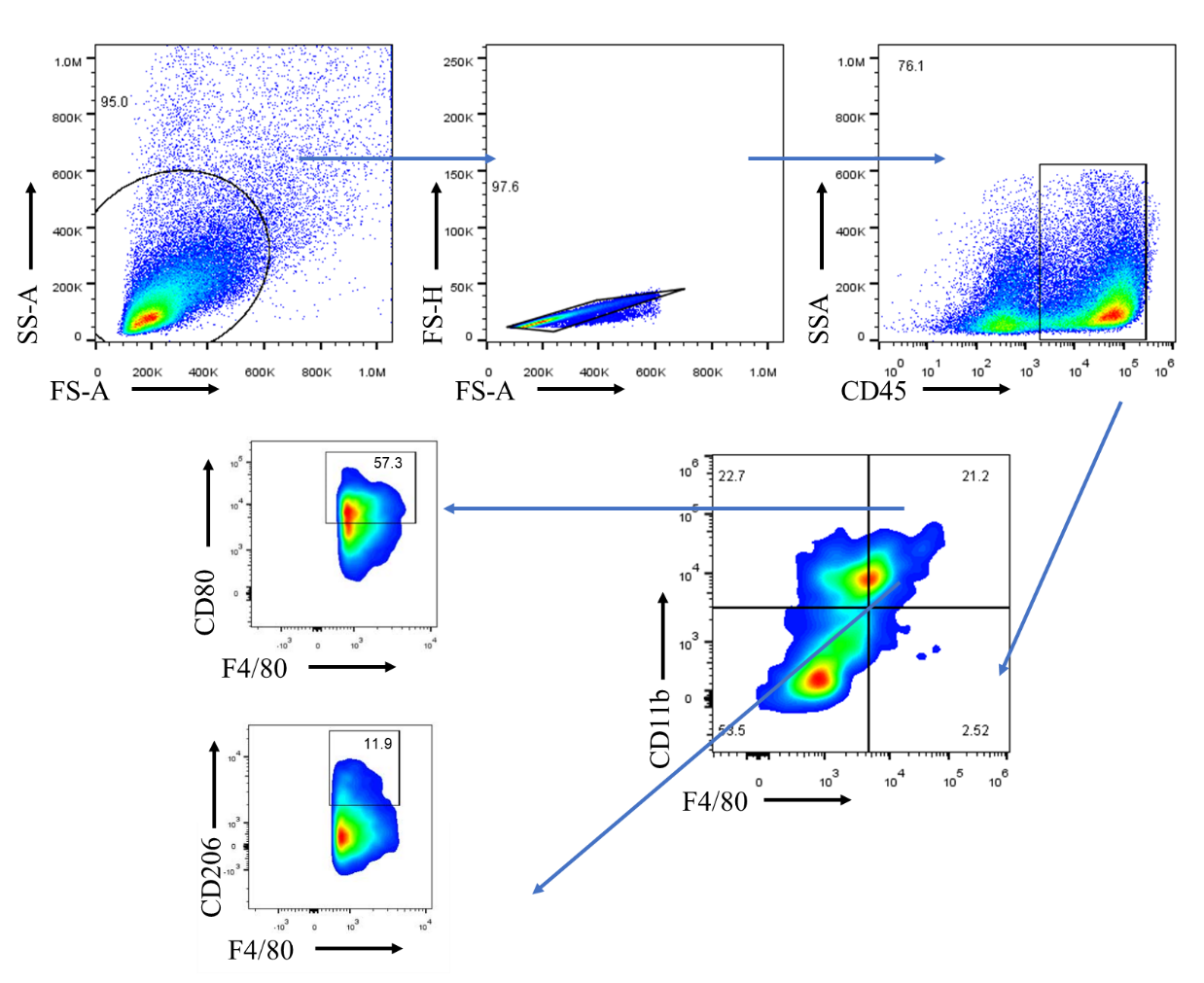


**Figure S14.** Gating strategies for M1 and M2 macrophages in BSG tumor models.


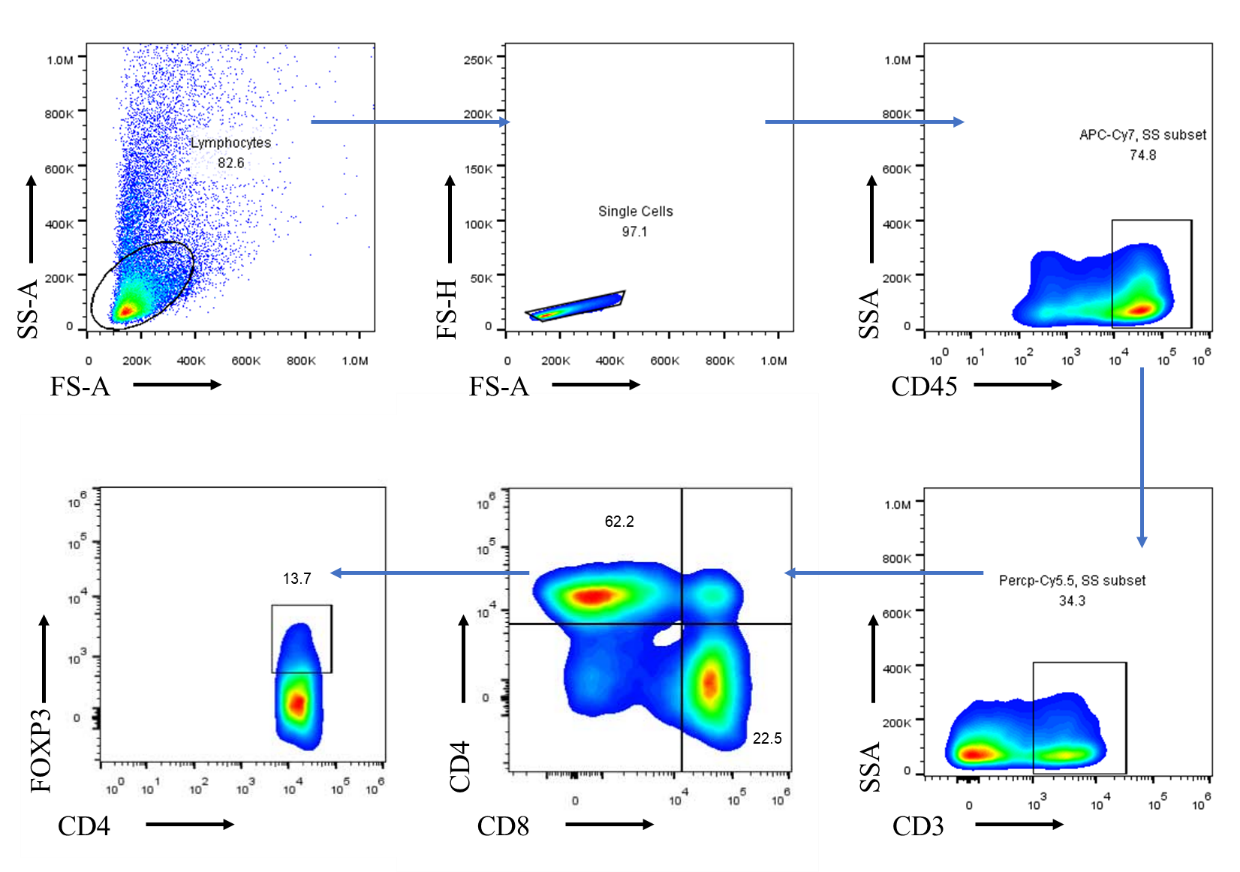


**Figure S15.** Gating strategies for CD8+ T cells, CD4+ T cells and Tregs in BSG tumor models.


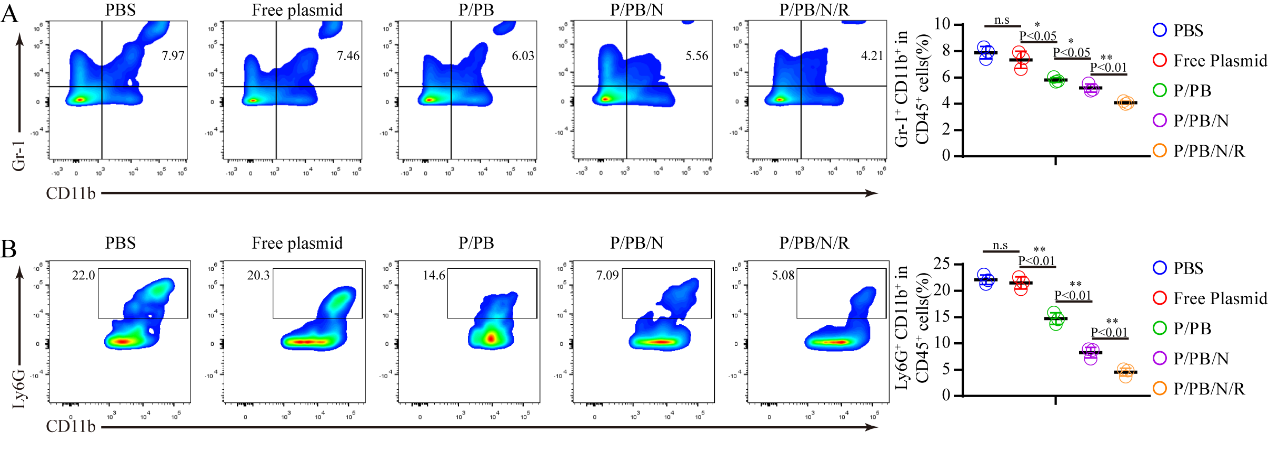


**Figure S16.** A) Representative flow cytometric analysis images (left) and relative quantification (right) of MDSCs (CD11b+Gr-1+) gating on CD45+ cells. Data are presented as the mean ± s.e.m. (n = 3). B) Representative flow cytometry analysis images and relative quantification of CD11b+ly6G+ neutrophil cells gating on CD45+ cells. Data are presented as the mean ± SEM (n = 3). Statistical significance was calculated via two-tailed Student’s t test. *P < 0.05; **P < 0.01.


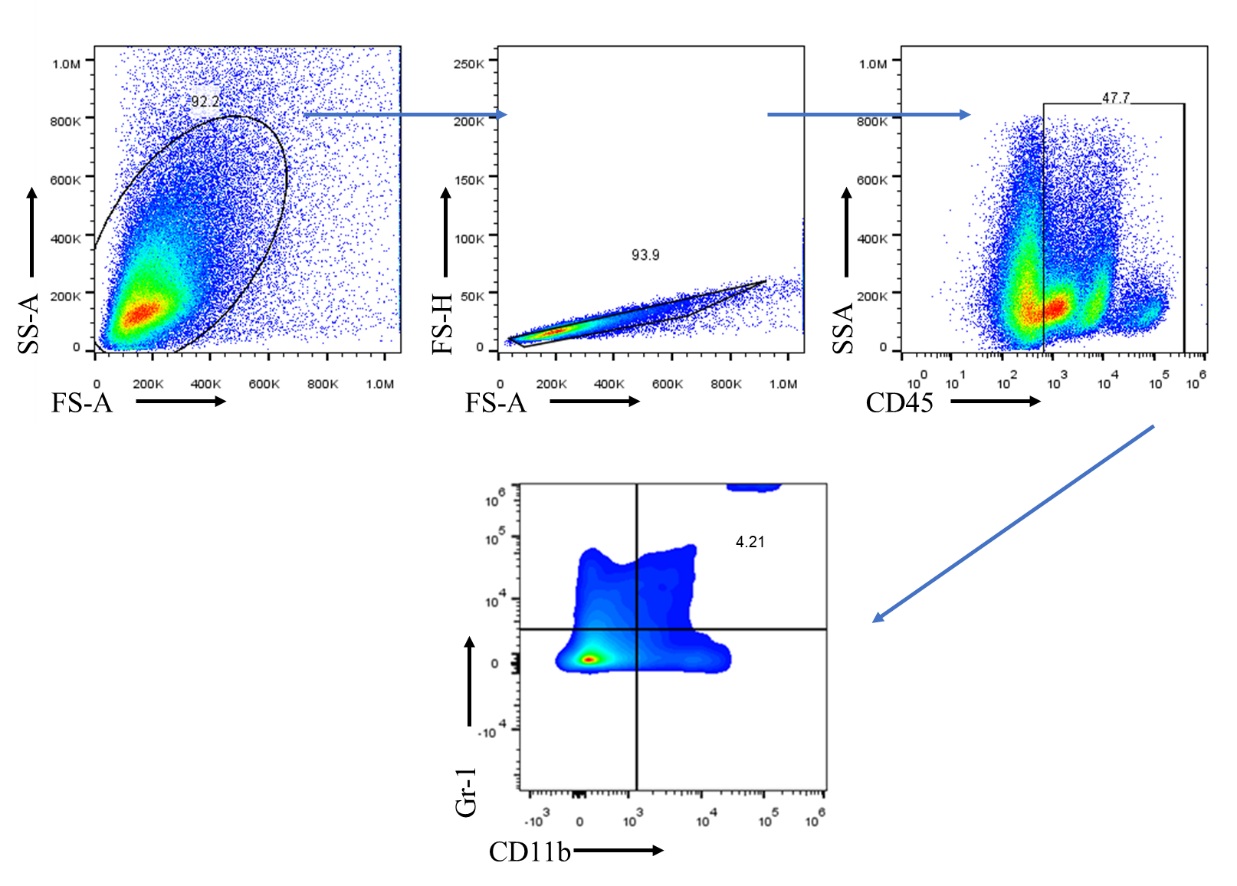


**Figure S17.** Gating strategies for MDSCs in BSG tumor models.


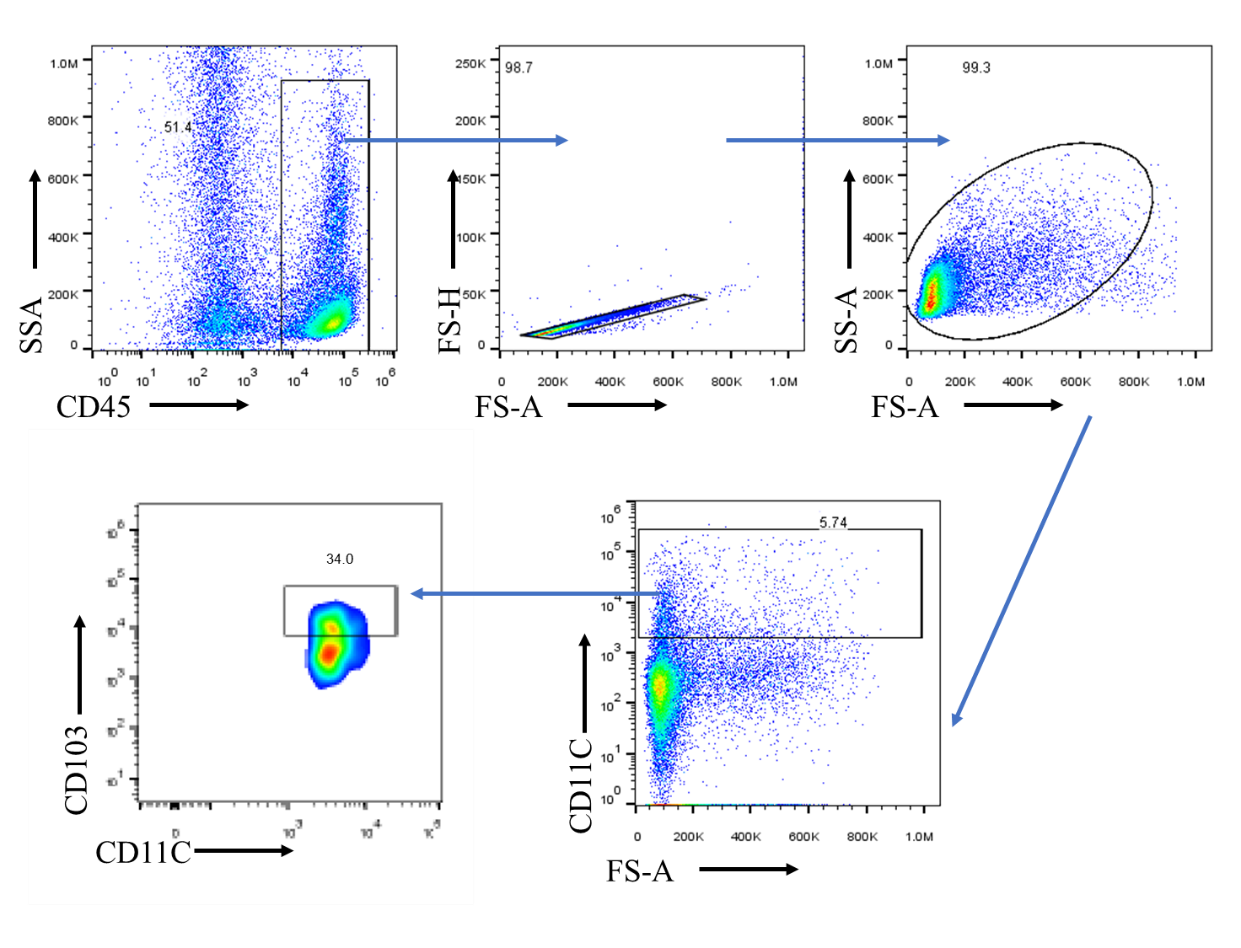


**Figure S18.** Gating strategies for mature dendritic cells in BSG tumor models.


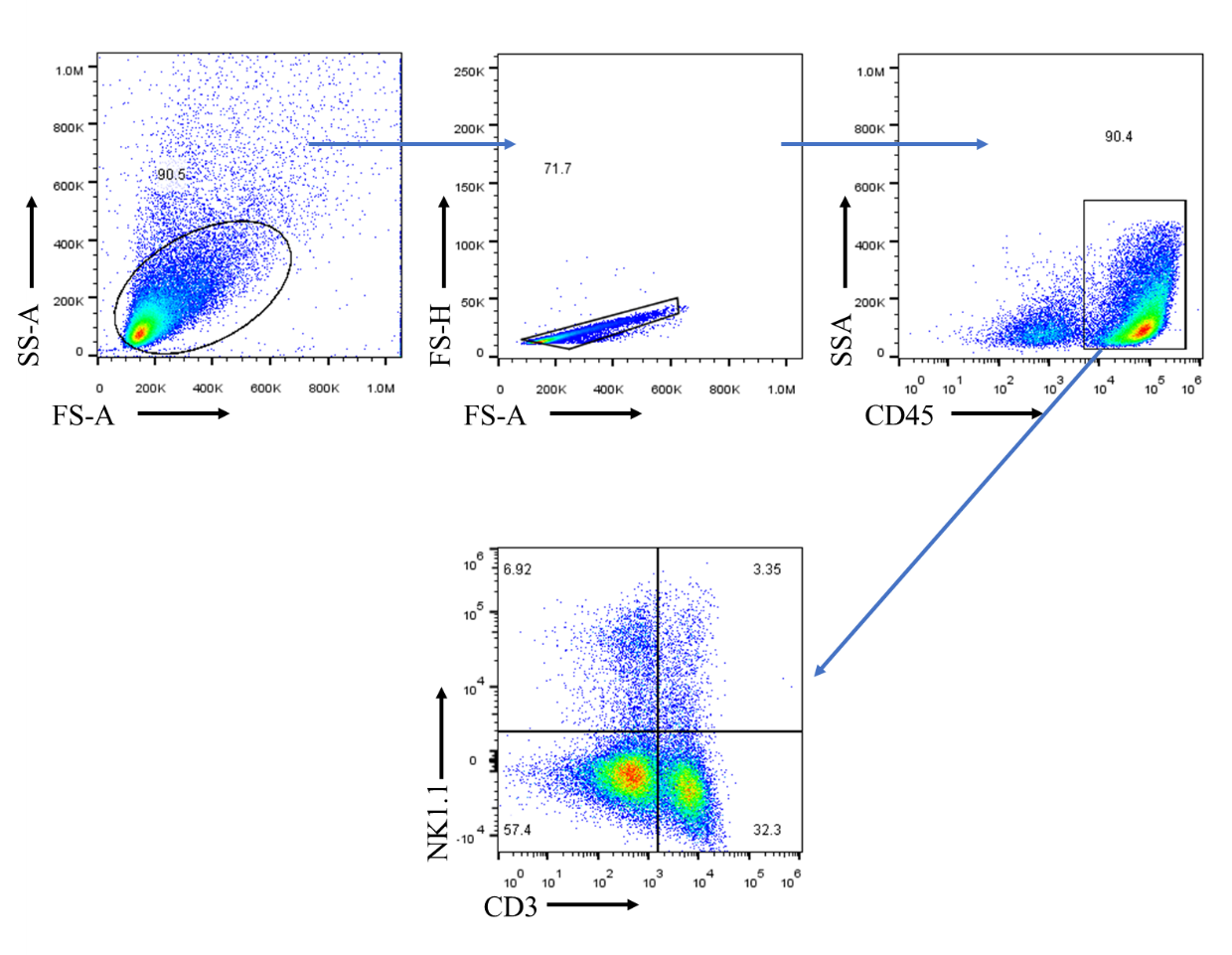


**Figure S19.** Gating strategies for nature killer cells in BSG tumor models.


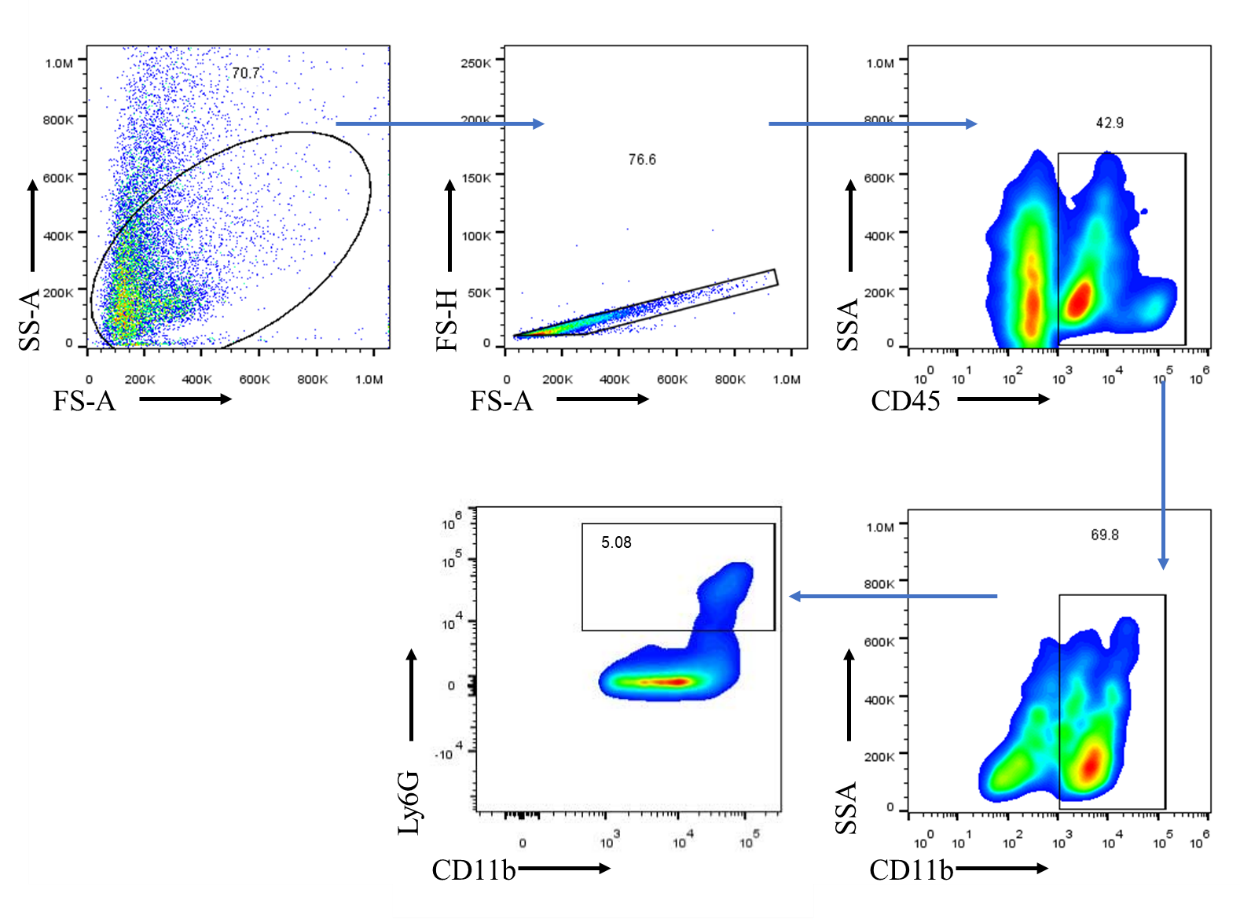


**Figure S20.** Gating strategies for neutrophils in BSG tumor models.


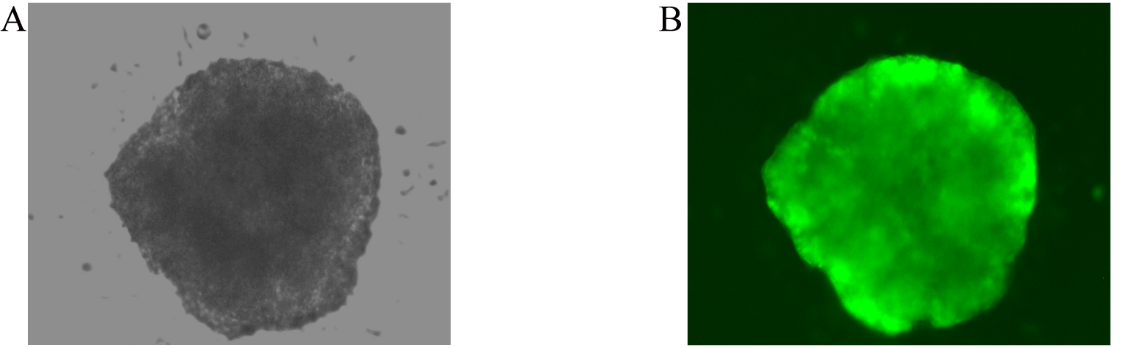


**Figure S21.** A) An image of the human luc+ EGFP+ brainstem cancer cell line in bright field and B) an image obtained under fluorescence microscope.


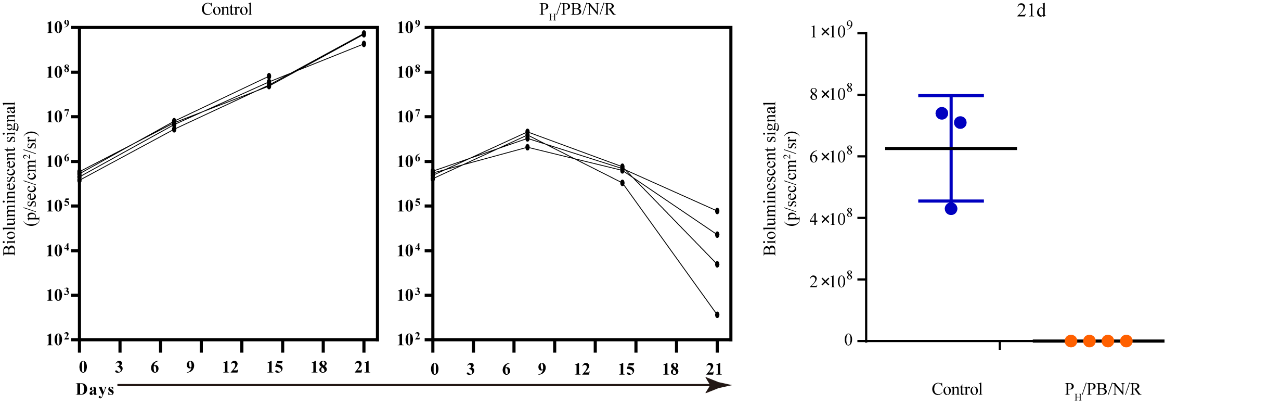


**Figure S22.** The quantitative fluorescence intensity of IVIS spectrum images in Figure 6C.


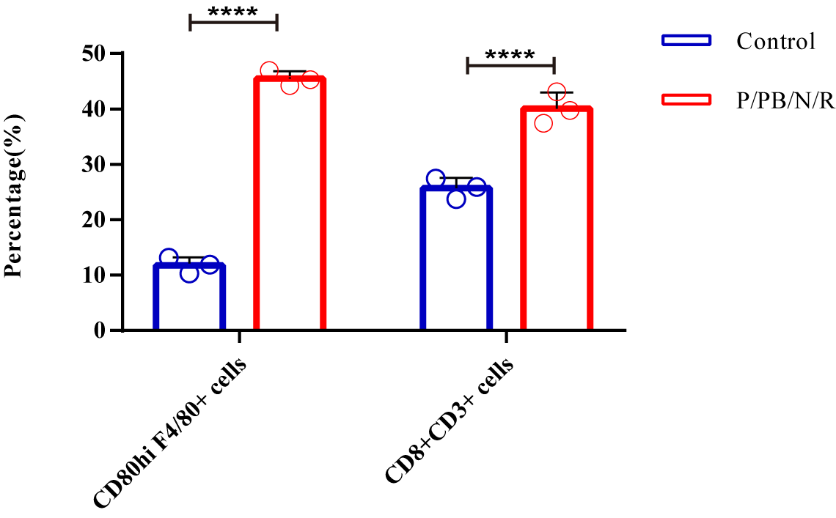


**Figure S23.** Relative quantification of CD80hiF4/80+CD11b+ cells and CD8+CD3+ cells gating on CD45+ cells. Data are presented as the mean ± SEM (n = 3). Statistical significance was calculated via a two-tailed Student’s t test. ****P < 0.0001.
